# Exploring the efficacy and molecular mechanism of Danhong injection comprehensively in the treatment of idiopathic pulmonary fibrosis by combining meta-analysis, network pharmacology, and molecular docking methods

**DOI:** 10.1097/MD.0000000000038133

**Published:** 2024-05-10

**Authors:** Xiaozheng Wu, Wen Li, Zhenliang Luo, Yunzhi Chen

**Affiliations:** a Department of Preclinical Medicine, Guizhou University of Traditional Chinese Medicine, Guiyang, China.

**Keywords:** Danhong, idiopathic pulmonary fibrosis, meta-analysis, network pharmacology

## Abstract

**Background:**

Danhong injection, a compound injection of Chinese herbal medicine, has been widely used in idiopathic pulmonary fibrosis (IPF) at present as an adjuvant treatment. However, the clinical efficacy and molecular mechanism of IPF are still unclear. This study will evaluate and explore the clinical efficacy and molecular mechanism of Danhong injection in the treatment of IPF.

**Methods:**

In meta-analysis, the computer was used to search 8 databases (PubMed, EMbase, CENTRAL, MEDLINE, CBM, CNKI, WanFang, and VIP) to collect the RCTs, and RevMan 5.3 and Stata 14.0 were used for statistical analysis. It has been registered on PROSPERO: CRD42020221096. In network pharmacology, the main chemical components and targets of the chemical components of Danhong injection were obtained in TCMSP and Swiss Target Prediction databases. The main targets of IPF were obtained through Gencards, Disgenet, OMIM, TTD, and DRUGBANK databases. The String platform was used to construct PPI networks. Cytoscape 3.8.2 was used to construct the “Danhong components – IPF targets-pathways” network. The molecular docking verification was conducted by Auto Dock.

**Results:**

Twelve RCTs were finally included with a total of 896 patients. The meta-analysis showed that Danhong injection could improve the clinical efficiency ([OR] = 0.25, 95% CI [0.15, 0.41]), lung function, arterial blood gas analysis, inflammatory cytokines, and serum cytokines associated with pulmonary fibrosis of IPF patients, respectively (*P* < .05). The core active components of Danhong injection on IPF were Luteolin, Quercetin, and Kaempferol, and the core targets were PTGS2, AR, ESR1, PPARG, and RELA. Danhong injection mainly improved IPF through PD-L1 expression and PD-1 checkpoint path in cancer, pathways in cancer, PI3K-Akt signaling pathway, etc.

**Conclusion:**

These results provided scientific basis for the clinical use of Danhong injection for the treatment of IPF, and provided a new direction to explore the potential mechanism of action of Danhong injection.

## 1. Introduction

Idiopathic pulmonary fibrosis (IPF) is an unexplained fibrotic interstitial pneumonia in which patients typically die within 2.5 to 5 years of definitive diagnosis.^[1]^ It is characterized by the accumulation of proliferative fibroblasts and myofibroblasts, and a large amount of matrix in lung interstitium as the focus, and abnormal expression of the main components of α-smooth muscle actin (α-SMA) and extracellular matrix (ECM: Collagen-I and Collagen-III).^[2]^ IPF is characterized by high morbidity and mortality and has become a serious health problem.^[3]^ The only approved drugs currently for the treatment of IPF are Pirfenidone and Nintedanib, however, these drugs can only delay the progression of IPF and do not improve the lesion.^[1,4]^ Therefore, it is necessary to develop other new drugs or alternative drugs to treat IPF.

IPF belongs to the category of “Pulmonary flaccidity” in traditional Chinese medicine (TCM).^[5]^ From the perspective of the pathogenesis of IPF, it is “blood stasis” essentially: blood stasis will lead to mutual resistance of sputum and stasis and eventually form IPF. Thus, blood stasis can be considered to be an important therapeutic target throughout the whole process of IPF.^[5]^ Danhong injection contains 2 TCMs: *Salvia miltiorrhiza* Bunge (the dried root and rhizome of *S miltiorrhiza*) and Carthami Flos (the dried flower of *Carthamus tinctorius* L.). All the above plant names have been checked with http://mpns.kew.org. *S miltiorrhiza* Bunge, named Danshen in Chinese, has the functions of dredging meridians and relieving pain; Carthami Flos, named Honghua in Chinese, also has the functions of unblocking meridians and relieving pain,^[6]^ and the combination of the 2 can complement each other, enhance the role of facilitating blood circulation and decreasing blood stasis. At present, as an adjuvant treatment to conventional treatment, Danhong injection has been widely used in cardiovascular disease,^[7]^ cerebrovascular disease,^[8]^ respiratory system disease,^[9]^ and kidney disease^[10]^ in China, and its curative effect is quite significant. In recent years, Danhong injection has also been widely used in different fibrosis fields.^[11–13]^ However, the clinical efficacy and molecular mechanism of its treatment of IPF are unclear.

This study combined meta-analysis, network pharmacology, and molecular docking to comprehensively evaluate and explore the clinical efficacy and molecular mechanism of Danhong injection in the treatment of IPF and provide strong evidence for it as a new alternative drug for IPF treatment.

## 2. Methods

### 2.1. Meta-analysis

This study has been registered on PROSPERO: CRD42020221096.

#### 2.1.1. Inclusion criteria

All included studies were randomized controlled trials (RCTs) of Danhong injection combining with conventional treatment for IPF, which must meet international^[1]^ or Chinese^[14]^ authoritative standards and be in English or Chinese. All patients were not restricted by gender, age, ethnicity, and nationality. Experimental group: combined use of Danhong injection on the basis of conventional treatment or control group. Control group: on the basis of conventional treatment, hormones, N-acetylcysteine (NAC), and other drugs were used. There were no restrictions on the dose, method of administration, and duration of the drugs in both groups. Conventional treatment included antibiotics and oxygen therapy, as well as other treatments.

#### 2.1.2. Exclusion criteria

Studies in which patients with other systemic serious diseases were excluded; non-RCTs, case reports, reviews, expert experience, and experimental studies were excluded; the results of the same study that has been published multiple times, only one of the most complete studies were included.

#### 2.1.3. Outcomes

Primary outcome: Clinical efficiency.^[15]^ Secondary outcomes: lung function; the analysis of arterial blood gas; inflammatory cytokines; serum cytokines associated with pulmonary fibrosis; adverse reactions.

#### 2.1.4. Literature search strategy

Eight databases were searched by using theme words and keywords: PubMed, EMbase, CENTRAL, MEDLINE, CBM, CNKI, WanFang, and VIP Chinese Science, and the search date was set to November 2, 2022. Relevant literature in Google Scholar is manually retrieved as a supplement at the same time. Search terms: “IPF”, or “Pulmonary fibrosis” or “Interstitial lung disease” or “Pulmonary interstitial fibrosis” or “IPF” and “Danhong” or “Danhong injection” and “Randomized controlled trial” or “RCT.” Table S1, Supplemental Digital Content, http://links.lww.com/MD/M461 shows the search strategies in PubMed.

#### 2.1.5. Literature screening and data extraction

The literatures were independently screened by 2 researchers (Wu and Li). Full-text readings were conducted for potentially eligible entries. Literatures were handed over to a third party (Chen) to decide whether to really include the statistics when there were different opinions after reading the full text. For literatures with incomplete data, the original author was contacted by email for details. The data tables were designed by following the principle of “PICOST” (participants, interventions, comparisons, outcomes, study design, and timing).

#### 2.1.6. Quality evaluation and bias risk assessment

The quality of the included studies and the risk of bias were assessed by using the modified Jadad Scale^[16]^ and Cochrane 5.1.0.

#### 2.1.7. Statistical analysis

In this study, all statistical analysis was performed in Revman 5.3 and Stata 14.0. The dichotomous variables were statistically analyzed by using odds ratio (OR) and 95% confidence interval (95% CI), and the continuous variables were statistically analyzed by using mean difference (MD) and 95% CI. When there was heterogeneity between the data (*P* < .1, *I*^2^ > 50%), the random effect model was used for analysis, on the contrary, the fixed-effect model was used. The Begg test and the Egger test were used to analyze publication bias. When there is obvious heterogeneity between the data, sensitivity analysis was conducted.

### 2.2. Network pharmacology and molecular docking

#### 2.2.1. Screening of targets of Danhong injection

In the traditional Chinese medicine system pharmacology (TCMSP) platform,^[17]^ the chemical components of *S miltiorrhiza* Bunge (Danshen) and Carthami Flos (Honghua) in Danhong injection were searched. The active ingredients were initially screened according to the ADME attribute values (oral bioavailability (OB) ≥ 30% and Drug-likeness (DL) ≥ 0.18), and the targets of these active ingredients were obtained in TCMSP. The 2D molecular structures of these active ingredients were entered into Swiss Target Prediction database^[18]^ and were screened at the same time to obtain the predicted targets of these components, and when the results display a higher probability value, it means a higher probability.

#### 2.2.2. Screening of targets of IPF

In the GeneCards database, disgenet database, OMIM database, TTD database, DRUGBANK database, “IPF” was entered and screened for potential targets for the treatment of IPF. According to the previous literature,^[19]^ higher score value in the Genecards and disgenet databases indicated that these targets were closely associated with the disease. Therefore, if there are too many targets, the targets whose Score was greater than the median were set as the potential targets for IPF. Finally, After merging the targets of 5 disease databases and removing duplicate values, potential targets for IPF were obtained.

#### 2.2.3. Establish PPI network for main targets of Danhong injection-IPF

The jvenn online platform^[20]^ was used to intersect the predicted targets of Danhong injection and the potential targets of IPF, and the Venny diagram was drawn at the same time, and then the intersection targets were submitted to the STRING11.5 database to establish the protein–protein interaction (PPI) network model^[21]^: the biological species was set to “*Homo sapiens*,” the minimum interaction threshold was set to “medium confidence > 0.4,” and the rest of the settings were the default settings. The intersection targets greater than the median degree value were selected and determined to be the main targets of Danhong injection acting on IPF according to the size of the degree value, and they were further submitted to the STRING11.5 database to obtain the PPI network of the main targets (the minimum interaction threshold was set to “highest confidence > 0.9”), and the PPI network was further analyzed through the Metascape platform to obtain the potential protein functional modules, and the function was described by analyzing the biological processes in which it participates.

#### 2.2.4. Enrichment analysis of targets functions and pathways of Danhong injection-IPF

With the aim of enrichment analysis of the main biological processes and signaling pathways of these targets, the main targets of Danhong injection acting on IPF were entered into the Metascape platform,^[22]^ and *P* < .01 was set at the same time, and finally the data results were saved and visualized by using the ehbio online platform. The enrichment analysis consisted of 4 parts: BP for biological processes of Danhong injection on IPF, MF for molecular functions, CC for cellular components, and KEGG for signaling pathways and the main targets contained in these pathways.^[23]^

#### 2.2.5. Construction of Danhong injection-IPF targets-signal pathway network diagram

CytoScape 3.8.2 was used to construct the network diagram of the main targets-pathway of Danhong injection-IPF, and the network topology parameters of the active ingredients and targets, including Degree, Betweenness, and Closeness, were analyzed by using the built-in tools of CytoScape3.8.2, and the core active ingredients, core targets and core pathways of Danhong injection in the treatment of IPF were judged according to the network topology parameters.

#### 2.2.6. Molecular docking verification

The top 5 targets in the network diagram of the main targets of Danhong injection-IPF were molecularly docked with the core active ingredients of Danhong injection: first, the 3D structure PDB format files of 5 target proteins were downloaded from the RSCB PDB database, and second, the SDF format files of the 2D structure of the core active ingredients of Danhong injection were downloaded from the PubChem database and converted into mol2 format files by using Open Babel GUI software. Molecular docking was carried out in Auto Dock software: first, the target proteins were dehydrated and hydrogenated. Second, Auto Dock software was used to convert the format files of Danhong injection’s core active ingredients and target proteins into PDBQT. Finally, run the Auto Dock program for molecular docking. If the binding energy is <0, it means that the ligands and the receptors can bind spontaneously.

## 3. Results

### 3.1. Meta-analysis

#### 3.1.1. Literature search results

**Figure 1.**
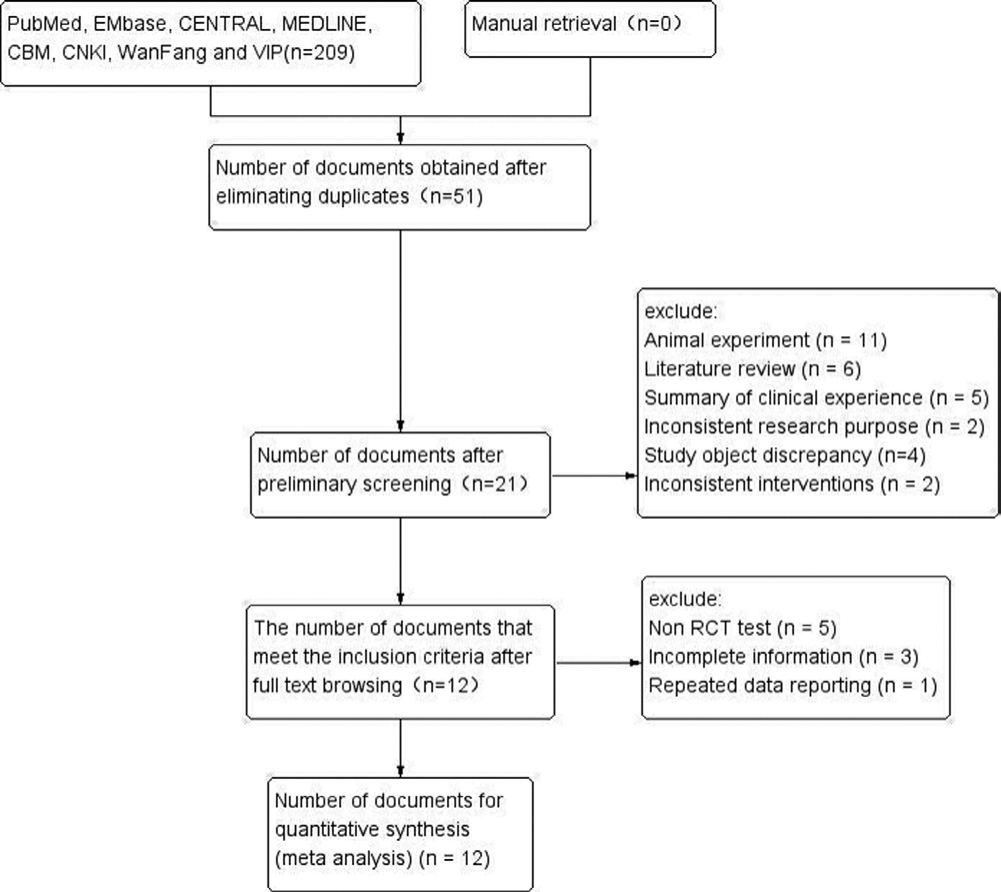
PRISMA literature screening flow diagram.

In meta-analysis, 209 relevant literature were screened initially, and 12 studies were finally included.^[24–35]^ A flowchart was developed according to the PRISMA statement^[36]^ (Fig. 1). All studies were conducted in China, and they contained a total of 896 patients (Danhong: 449; Control:447). Table 1 shows the specific data characteristics of all included studies.

**Table 1:** Basic features of the included studies.

Studies	Sample (n)		Gender (male/female) (n)	Age (yr)	Average course of disease (yr)	Outcomes	Course (wk)	Adverse reactions	Interventions	
	E	C							Experimental	Control
Cai^[24]^	30	30	E:20/10 C:21/9	E:69.1 C:65.8	E:3.1 C:2.8	PaO2	6	E:3/30; C:3/30. There were 3 cases in the experimental group and 3 cases in the control group. After adjusting to Erythromycin Tablets, they disappeared after taking medicine after meal (more consideration of erythromycin gastrointestinal stimulation), and no obvious adverse reactions were found.	Danhong injection, Erythromycin Tablets, routine treatment	Erythromycin tablets, routine treatment
Chen^[25]^	45	45	E:27/18 C:25/20	E:50.2 ± 9.6 C:49.5 ± 8.7	E:4.8 ± 2.1 C:4.6 ± 1.8	FVC, DLCO, PaO2	12	Not described	Danhong injection, Prednisolone, routine treatment	Prednisolone, routine treatment
Li^[26]^	34	34	E:11/13 C:14/10	E:56.32 ± 3.29 C:56.38 ± 3.27	–	Clinical efficiency, DLCO, PaO2	8	Not described	Danhong injection, Prednisone, routine treatment	Prednisolone, routine treatment
Lin^[27]^	35	35	E:20/15 C:20/15	E:62. 41 ± 9. 82 C:61.53 ± 12.52	E:2.54 ± 1.71 C:2.85 ± 1.62	FEV1/FVC%, DLCO, PaO2, HA, ColIII, PCIII, LN, BUN	12	Non	Danhong injection, Methylprednisolone, routine treatment	Methylprednisolone, routine treatment
Ren^[28]^	24	24	–	E:50.63 ± 10.63 C:50.86 ± 10.22	E:6.07 ± 3.34 C:6.36 ± 3.22	Clinical efficiency, FVC, DLCO, PaO2	6	Not described	Danhong injection, Prednisolone, routine treatment	Prednisolone, routine treatment
Sun^[29]^	35	32	E:19/16; C:18/14	E:48.2 ± 16.3 C:50.8 ± 17.4	E:4.6 ± 2.8 C:4.6 ± 2.7	Clinical efficiency, DLCO, PaO2, TNF-α, TGF-β1	12	Not described	Danhong injection, Edaravone, routine treatment	Edaravone, routine treatment
Wang^[30]^	25	25	E:14/11 C:13/12	E: 36-58 C:36-67	–	Clinical efficiency, DLCO, PaO2	6	Not described	Danhong injection, Prednisolone, routine treatment	Prednisolone, routine treatment
Wang^[31]^	60	60	E:28/32; C:26/34	E: 64.0 ± 5.7 C:63.0 ± 6.2	E:6.3 ± 1.8 C:5.9 ± 2.7	FVC, PaO2, TGF-β1, HA, PCIII, LN	12	Not described	Danhong injection, Acetylcysteine, routine treatment	Acetylcysteine, routine treatment
Wu^[32]^	30	30	E:18/12 C:19/11	E:61.56 ± 12.33 C:61.80 ± 12.18	E:2.88 ± 1.56 C:2.52 ± 1.60	DLCO, PaO2, HA, PCIII, LN	12	Non	Danhong injection, Methylprednisolone, routine treatment	Methylprednisolone, routine treatment
Yin^[33]^	41	41	E:20/21 C:19/22	E: 52 C: 55	–	Clinical efficiency, DLCO, PaO2	6	Not described	Danhong injection, Prednisolone, routine treatment	Prednisolone, routine treatment
Zhao^[34]^	40	40	E:26/14 C:27/13	E:62.3 ± 8.9 C:62.8 ± 8.7	E:2.8 ± 1.6 C:2.8 ± 1.5	FEV1/FVC%, DLCO, PaO2, TNF-α, TGF-β1, HA, ColIII, PCIII, LN, BUN	12	Not described	Danhong injection, Acetylcysteine, routine treatment	Acetylcysteine, routine treatment
Zhou^[35]^	50	51	E:28/22 C:27/24	E:55.31 ± 9.36 C:54.96 ± 9.72	E:4M-2Y C:3M-2Y	Clinical efficiency	8	Not described	Danhong injection, Prednisone, Telmisartan, routine treatment	Prednisone, Telmisartan, routine treatment

C = control group, E = experimental group.

#### 3.1.2. Quality evaluation and bias risk assessment

Data from all 12 RCTs were complete, and they all used randomization methods. These RCTs all described the comparability of baseline data between the experimental and control groups in detail, and they all described the therapeutic measures and efficacy outcomes in detail. However, they did not specify the method of allocation concealment or blinding. The evaluation results of the Jadad scale^[16]^ are shown in Table S2, Supplemental Digital Content, http://links.lww.com/MD/M462: 8 studies were with 2 points (low-quality) and 4 studies were with 3 points (medium-quality).^[24,25,29,32]^ The low and medium risks resulting from random sequences accounted for 32% and 68% of the selection bias of the original study, respectively (Figures S1 and S2, Supplemental Digital Content, http://links.lww.com/MD/M445, http://links.lww.com/MD/M446). Therefore, there are certain biases in the included studies, such as selection, implementation, and measurement bias.

#### 3.1.3. Primary outcome (clinical efficacy)

**Figure 2.**
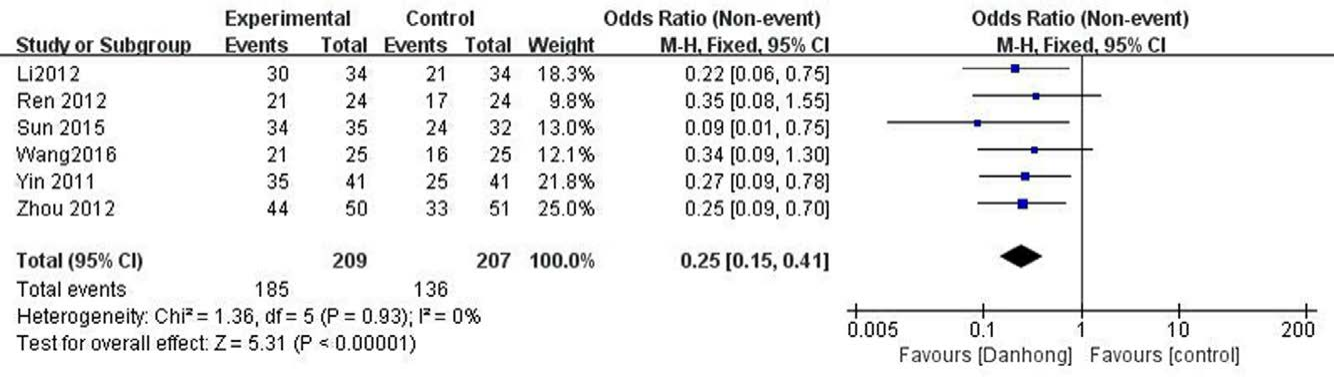
The forest plot of clinical efficacy.

There were 6 trials^[26,28–30,33,35]^ reported clinical efficacy. As there was no significant heterogeneity between studies, the fixed-effect model was used for analysis (*P* = .93, *I*^2^ = 0%). As shown in Figure 2, the clinical efficacy of the Danhong injection group was higher than that of the control group ([OR] = 0.25, 95% CI [0.15, 0.41], *P* < .00001).

#### 3.1.4. Secondary outcomes

Danhong injection could better improve the secondary outcomes of IPF patients compared with conventional treatment (Table 2 and Figures S3–S13, Supplemental Digital Content, http://links.lww.com/MD/M447, http://links.lww.com/MD/M448, http://links.lww.com/MD/M449, http://links.lww.com/MD/M450, http://links.lww.com/MD/M451, http://links.lww.com/MD/M452, http://links.lww.com/MD/M453, http://links.lww.com/MD/M454, http://links.lww.com/MD/M455, http://links.lww.com/MD/M456, http://links.lww.com/MD/M457): lung function: FEV_1_/FVC%: (MD = 12.25, 95% CI [9.60, 14.89], *P* < .00001); FVC(L): ([MD] = 0.28, 95% CI [0.21, 0.35], *P* < .00001). The analysis of arterial blood gas: DLCO (mL/(min mm Hg)): ([MD] = 4.58, 95% CI [3.57, 5.60], *P* < .00001); PaO2 (mm Hg): ([MD] = 14.16, 95% CI [12.74, 15.59], *P* < .00001). Inflammatory cytokines: TNF-α (pg/L): ([MD] = −8.37, 95% CI [−10.81, −5.92], *P* < .00001); TGF-β_1_ (pg/L)_:_ ([MD] = −4.67, 95% CI [−6.65, −2.70], *P* < .00001). Serum cytokines associated with pulmonary fibrosis: HA (μg/L): (MD = −28.81 [−48.52, −9.11], .004); LN (μg/L): (MD = −36.52, [−49.71, −23.32], <.00001); PCIII (μg/L): (MD = −15.55, [−19.73, −11.36], <.00001); ColIII (mg/L): (MD = −35.18, [−41.37, −29.00], <.00001); BUN (mmol/L): (MD = −4.90, [−6.16, −3.64], <.00001).

**Table 2:** The results of meta-analysis of secondary outcomes.

Outcomes	Index	Studies (n)	Heterogeneity		Cases (n)		Model	Effect size	MD and 95%CL	Effect *P* value
			*P* value	*I*^2^ (%)	Experimental	Control				
Lung function	FEV1/FVC%	2	.42	0	75	75	Fixed	MD	12.25 [9.60, 14.89]	<.00001
	FVC (L)	3	.40	0	129	129	Fixed	MD	0.28 [0.21, 0.35]	<.00001
The analysis of arterial blood gas	DLCO (mL/(min mm Hg))	9	.008	61	309	306	Random	MD	4.58 [3.57, 5.60]	<.00001
	PaO2 (mm Hg)	11	<.00001	84	399	396	Random	MD	14.16 [12.74, 15.59]	<.00001
Inflammatory cytokines	TNF-α (pg/L)	2	.78	0	75	72	Fixed	MD	−8.37 [−10.81, −5.92]	<.00001
	TGF-β1 (pg/L)	3	.007	80	135	132	Random	MD	−4.67 [−6.65, −2.70]	<.00001
Serum cytokines associated with pulmonary fibrosis	HA (μg/L)	4	<.00001	90	165	165	Random	MD	−28.81 [−48.52, −9.11]	.004
	LN (μg/L)	4	.01	73	165	165	Random	MD	−36.52 [−49.71, −23.32]	<.00001
	PCIII (μg/L)	4	.14	45	165	165	Fixed	MD	−15.55 [−19.73, −11.36]	<.00001
	ColIII (mg/L)	2	.43	0	75	75	Fixed	MD	−35.18 [−41.37, −29.00]	<.00001
	BUN (mmol/L)	2	.41	0	75	75	Fixed	MD	−4.90 [−6.16, −3.64]	<.00001

#### 3.1.5. Adverse reactions

One study^[24]^ reported gastrointestinal discomfort in 3 patients in the Danhong injection group, but this was mainly due to the use of erythromycin. There were no obvious adverse reactions in other patients. In addition, 2 studies^[27,32]^ reported no adverse reactions and the other 9 studies did not describe adverse reactions.

#### 3.1.6. Publication bias and sensitivity analysis

**Figure 3.**
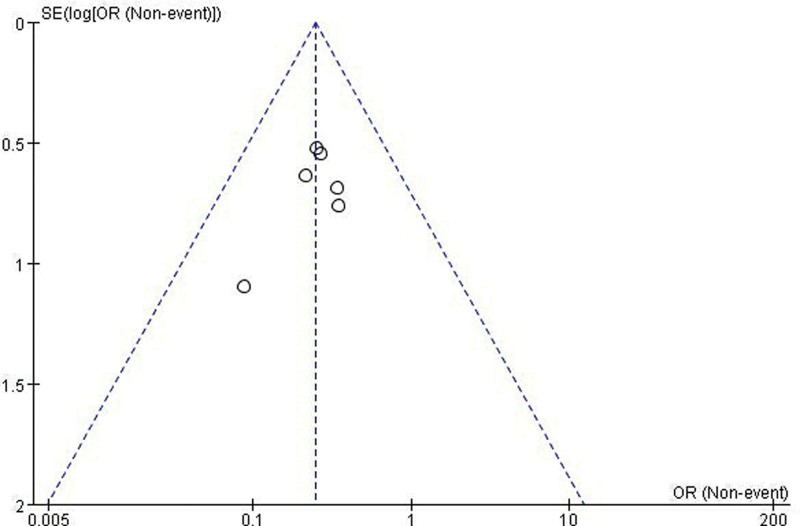
The funnel plot of clinical efficacy.

The funnel plot of clinical efficacy was symmetrical (Fig. 3), and there was no publication bias (*P*_Begg_ = 1.000; *P*_Egger_ = 0.298) (Tables S3 and S4, Supplemental Digital Content, http://links.lww.com/MD/M463, http://links.lww.com/MD/M464 and Figures S14 and S15, Supplemental Digital Content, http://links.lww.com/MD/M458, http://links.lww.com/MD/M459). In addition, the results of the sensitivity analysis had a minimum value of not <1, indicating good stability of the results after excluding either study (Table S5, Supplemental Digital Content, http://links.lww.com/MD/M465 and Figure S16, Supplemental Digital Content, http://links.lww.com/MD/M460).

### 3.2. Network pharmacology

#### 3.2.1. Target acquisition of active components of Danhong injection

Two hundred two chemical components of *S miltiorrhiza* Bunge (Danshen) and 189 chemical components of Carthami Flos (Honghua) were preliminarily extracted. After ADME screening, 65 active components of *S miltiorrhiza* Bunge (Danshen) and 22 active components of Carthami Flos (Honghua) were obtained, and a total of 84 active components of Danhong injection were obtained after combining them (Table S6, Supplemental Digital Content, http://links.lww.com/MD/M466). There were 349 targets of active components of *S miltiorrhiza* Bunge (Danshen) and 296 targets of active components of Carthami Flos (Honghua). After the combination, a total of 476 targets of Danhong injection were obtained by deleting the duplicate values.

#### 3.2.2. Acquisition of IPF-related targets

The targets obtained in 5 databases were combined, and the duplicate values were removed, and 1870 IPF-related targets were finally obtained. As shown in Table S7, Supplemental Digital Content, http://links.lww.com/MD/M467.

#### 3.2.3. PPI network construction of Danhong injection-IPF targets

**Figure 4.**
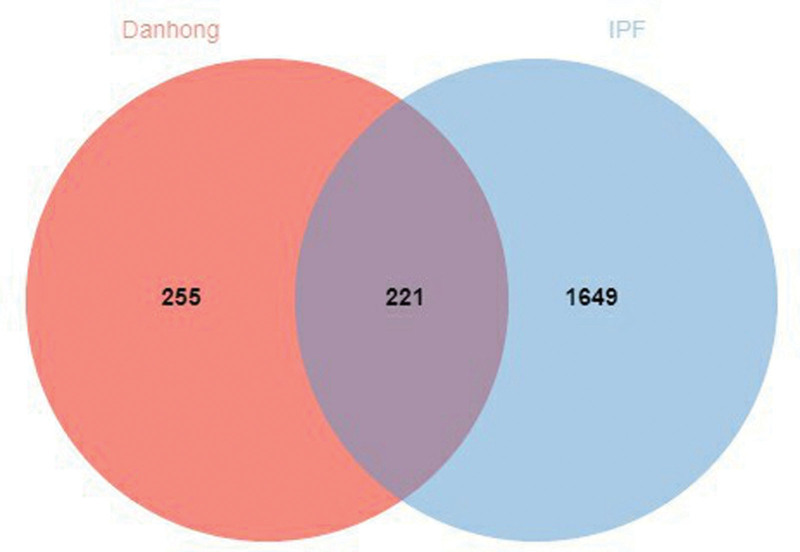
Venny diagram of the targets of Danhong injection-IPF (jvenn online tool, Designed by GenoToul Bioinfo and Sigenae teams.).

**Figure 5.**
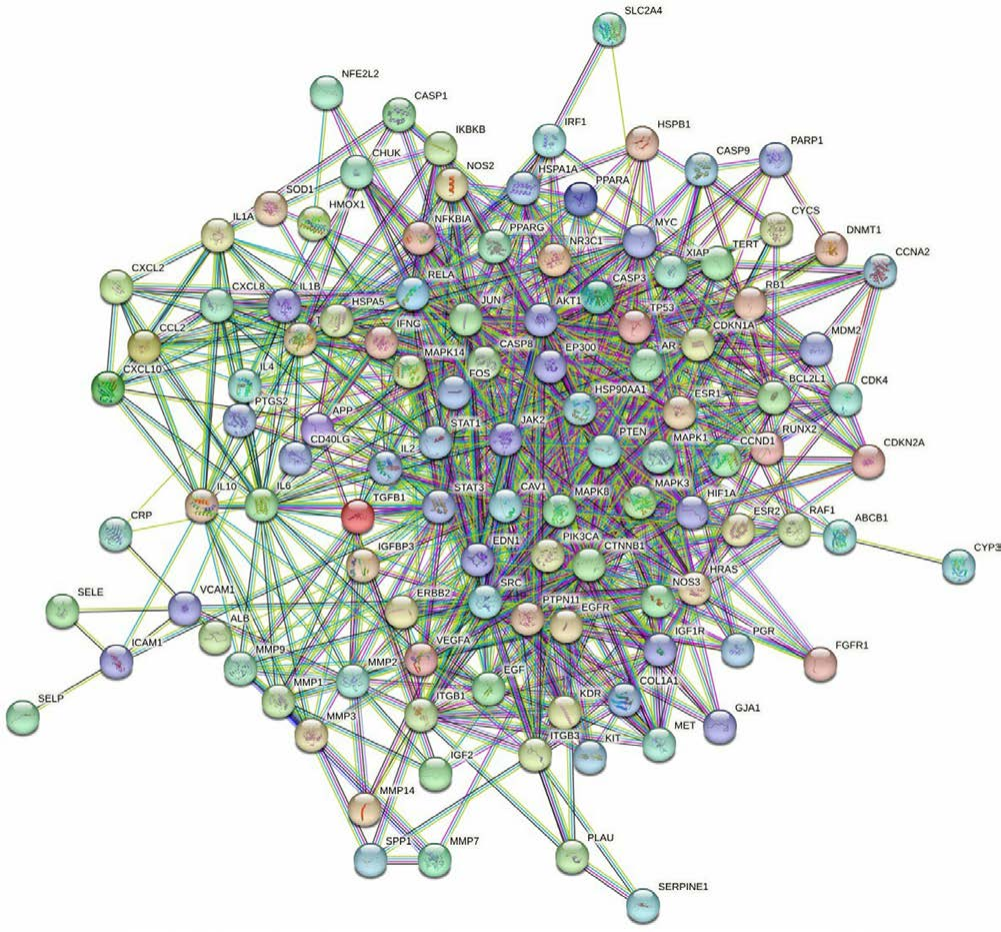
PPI network of Danhong injection-IPF targets (STRING online tool, version 11.5, Supported by STRING CONSORTIUM 2023).

**Figure 6.**
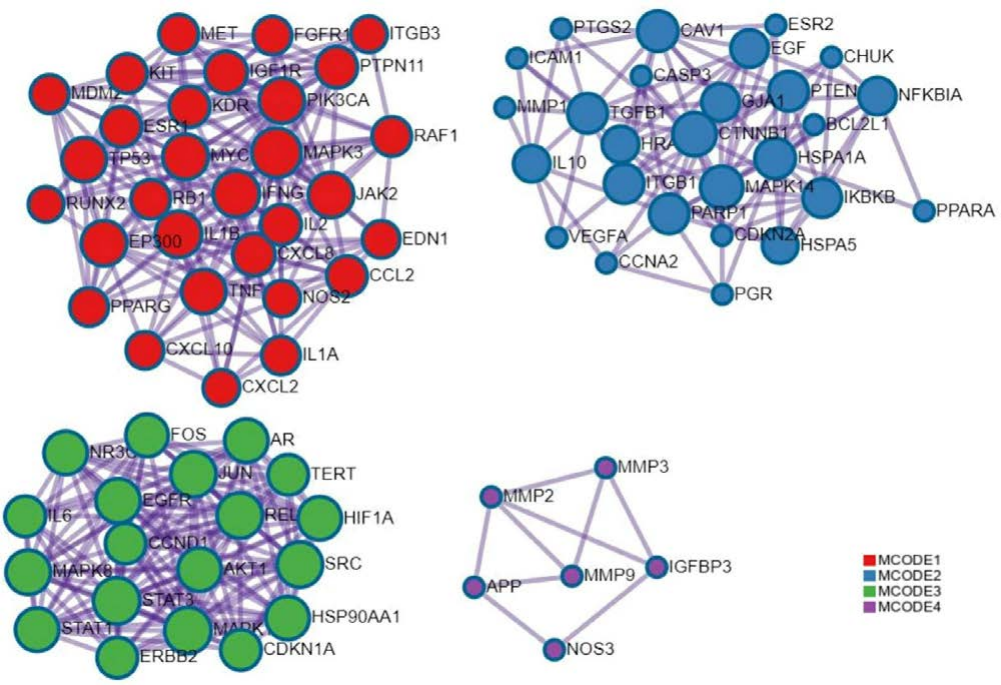
Module in PPI network of Danhong injection – IPF targets (Metascape data platform, Supported by National Institutes of Health (NIH) grants U19 AI106754; U19 AI135972; R01 DA03373).

The 476 targets of the screened Danhong injection were intersected with the 1870 disease targets of IPF, and 221 intersecting targets were obtained, as shown in Figure 4. Furthermore, 221 intersecting targets were submitted to the STRING11.5 platform, and the degree values of these 221 intersecting targets were calculated according to the node degrees module in the platform, and 114 targets greater than the median (≧42) were set as the main targets of Danhong injection acting on IPF according to the median of the degree values. After obtaining the main targets, they were further submitted to the STRING11.5 platform and the PPI network diagram of the main targets of Danhong injection acting on IPF was obtained (Fig. 5). The interaction relationship of these main targets was analyzed by molecular complex detection algorithm to obtain the modules in PPI networks^[37]^ in Metascape (Fig. 6), and the biological processes that retain the 3 best scores in the Modules according to the *P* values were described functionally (Table 3).

**Table 3:** Function description of PPI network of Danhong injection – IPF targets.

GO	Description	Log10(P)
GO:0009725	Response to hormone	−52.4
GO:0030335	Positive regulation of cell migration	−51.6
GO:2000147	Positive regulation of cell motility	−50.7

#### 3.2.4. Enrichment analysis of target functions and pathways

**Figure 7.**
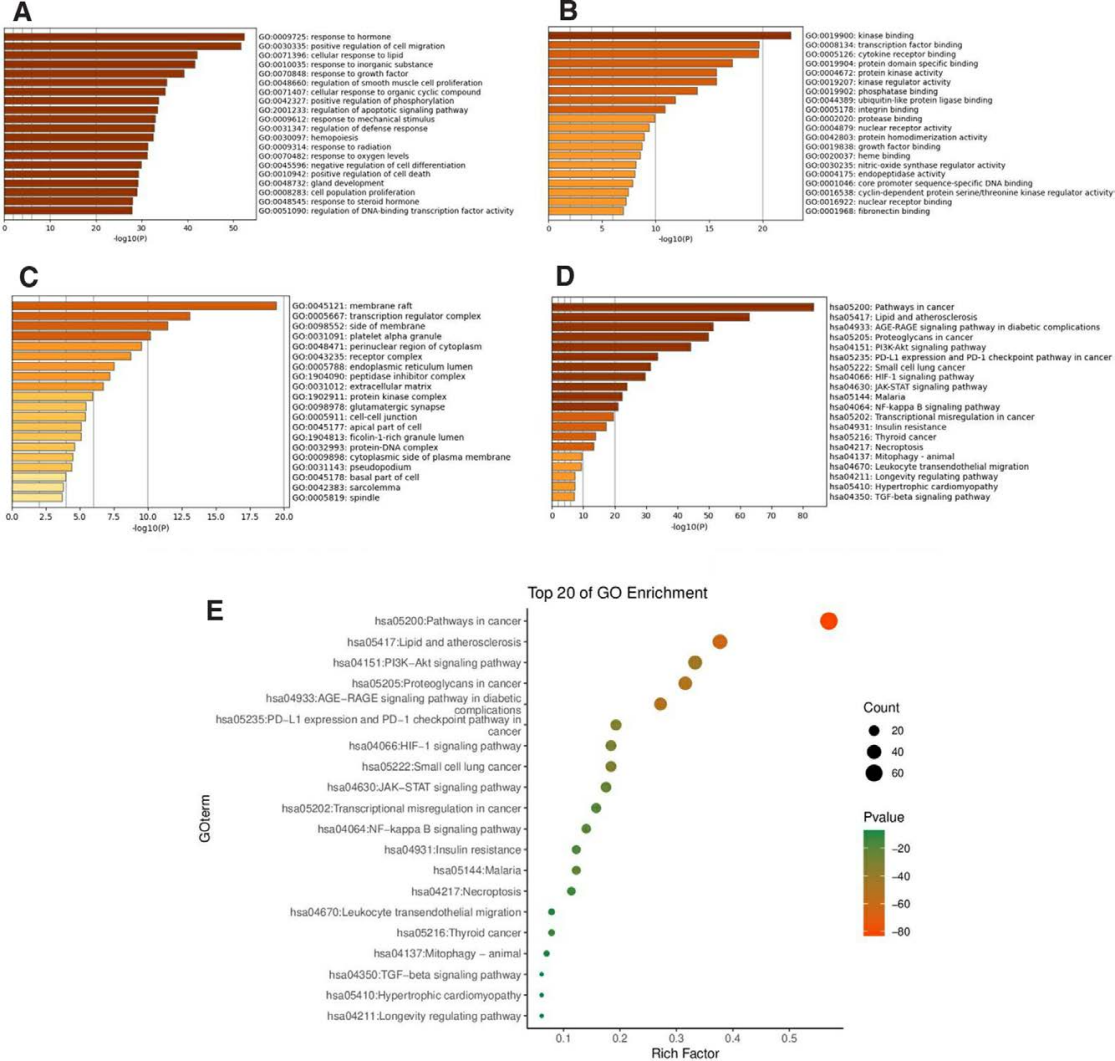
Enrichment analysis diagram (Metascape data platform, Supported by National Institutes of Health (NIH) Grants U19 AI106754; U19 AI135972; R01 DA03373; EhBIO’s online platform, version 1.0, Supported by Beijing Internet Content Provider (ICP) No. 15041106-1). (A) Go-BP analysis. (B) GO-MF analysis. (C) GO-CC analysis. (D) KEGG analysis. (E) Bubble diagram of KEGG analysis.

The Metascape was used to perform functional and pathway analysis of 114 main targets of Danhong injection acting on IPF, and the results were visualized with the help of the ehbio online platform. The results showed that the main biological processes of Danhong injection on IPF included response to hormone, positive regulation of cell migration, cellular response to lipid, response to inorganic substance, and response to growth factor, etc (Fig. 7A). The main molecular functions included kinase binding, transcription factor binding, cytokine receptor binding, protein domain specific binding, and protein kinase activity, etc (Fig. 7B). The main cellular components included membrane raft, transcription regulator complex, side of membrane, platelet alpha granule, perinuclear region of cytoplasm, etc (Fig. 7c). The main signaling pathways included Pathways in cancer (PC), lipid and atherosclerosis (LA), AGE-RAGE signaling pathway in diabetic complications (ARPDC), proteoglycans in cancer (PRC), PI3K-Akt signaling pathway (PASP), etc (Fig. 7D and E), and more detailed enrichment results are shown in Table S8, Supplemental Digital Content, http://links.lww.com/MD/M468.

#### 3.2.5. Construction of the main components of Danhong injection – IPF targets – pathways network diagram

**Figure 8.**
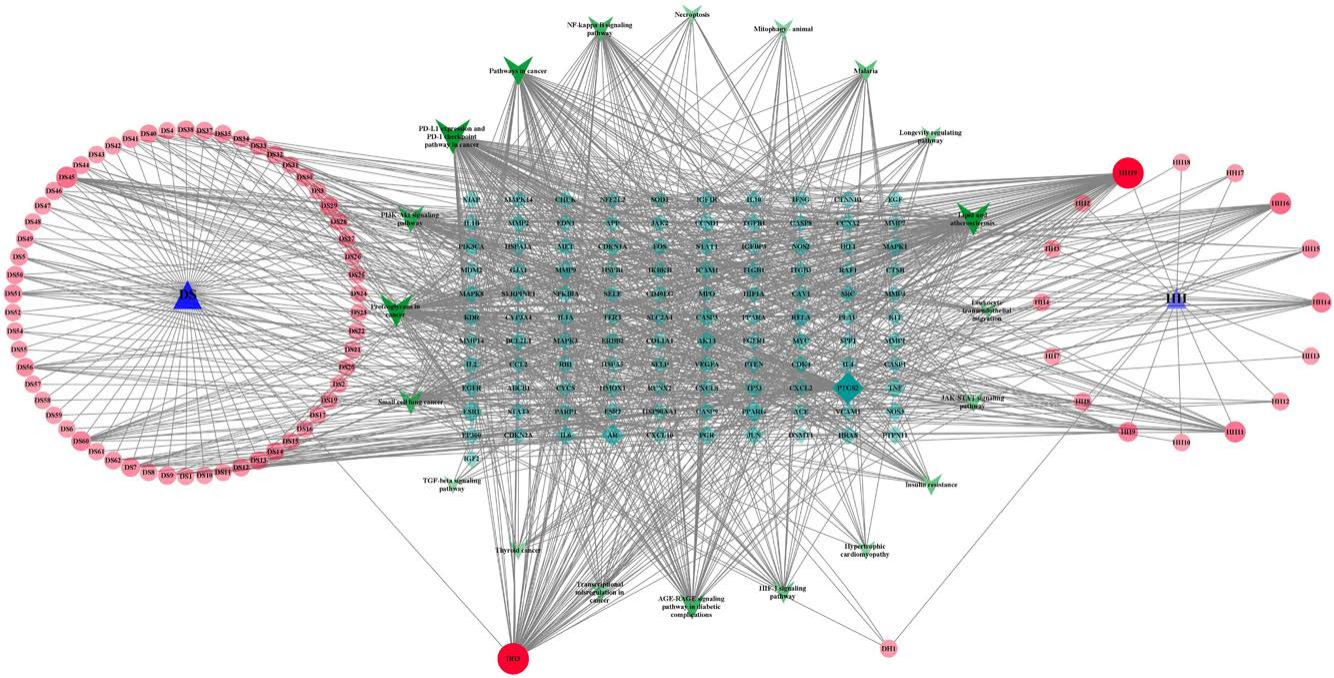
Danhong injection components-IPF targets-pathways network diagram (DS: Danshen (*S miltiorrhiza* Bunge); HH: Honghua (Carthami Flos); DH: Danshen (*S miltiorrhiza* Bunge) and Honghua (Carthami Flos)) (CytoScape, version 3.8.2, provided by the U.S. National Institute of General Medical Sciences (NIGMS) R01 GM070743).

Twenty major pathways and related targets were introduced into CytoScape3.8.2 to construct the network diagram of the main component of Danhong injection-IPF main target-pathway, as shown in Figure 8. The network topology parameters of Danhong injection acting on IPF were analyzed by the built-in Network Analyzer of CytoScape3.8.2, and the core components, core targets, and core signaling pathways of Danhong injection acting on IPF were obtained. The results showed that Luteolin had a Degree of 74, Betweenness Centrality of 0.0338, and Closeness Centrality of 0.4592, therefore Luteolin was predicted to be the core component of Danhong injection to improve IPF, followed by Quercetin and Kaempferol, as shown in Table 4. The results showed that PTGS2 had a Degree of 75, Betweenness Centrality of 0.29, and Closeness Centrality of 0.5714 in the network, therefore it is predicted that PTGS2 was the core target of Danhong injection on IPF. The remaining targets ranked by Degree value were AR (31), ESR1, PPARG (26), RELA (21), which were relatively important core targets, as shown in Table 5. The results showed that PD-L1 expression and PD-1 checkpoint pathway in cancer (PPCPC) had a Degree of 92, Betweenness Centrality of 0.1465, and Closeness Centrality of 0.6029, thus PPCPC were predicted to be the most core signaling pathways of Danhong injection acting on IPF. The remaining pathways were ranked according to Degree value: LA (84), PRC (81), PC (65), PASP (50), which are relatively important core signaling pathways, as shown in Table 6.

**Table 4:**
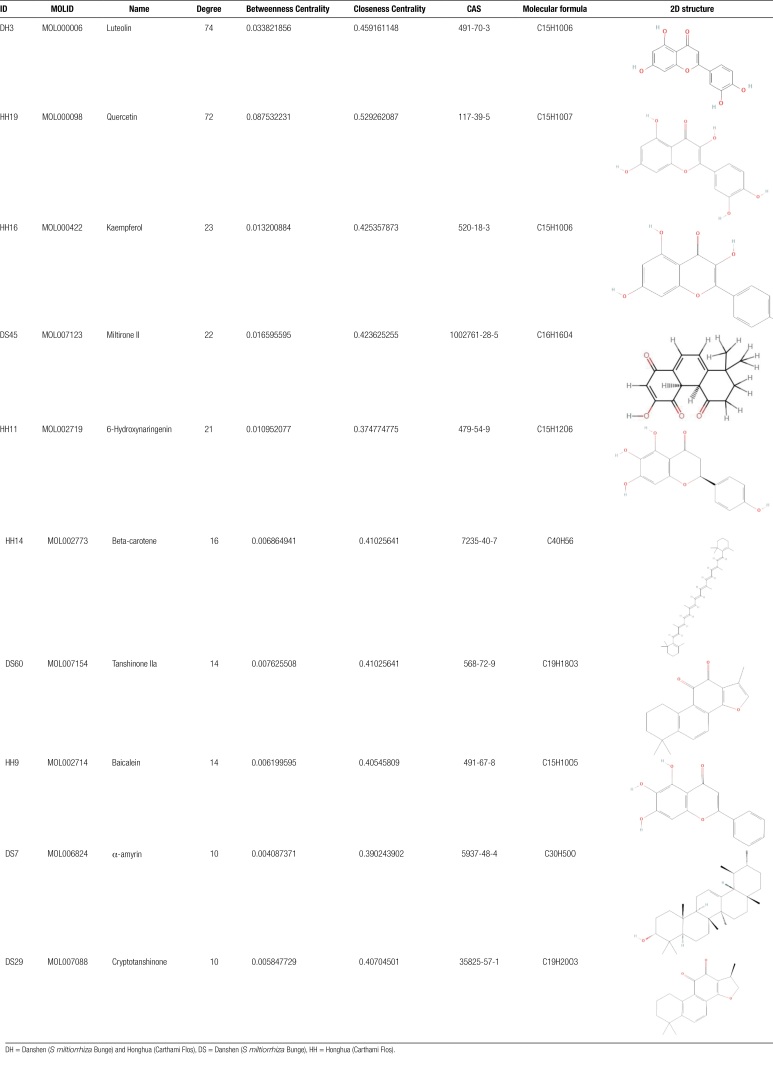
Characteristic parameters of network nodes of main active components of Danhong injection (Top 10).

**Table 5:** Characteristic parameters of target nodes of main active components of Danhong injection (Top 14).

Targets	Degree	Betweenness Centrality	Closeness centrality
PTGS2	75	0.290024781	0.571428571
AR	31	0.032165043	0.460176991
ESR1	26	0.026796576	0.440677966
PPARG	26	0.019842712	0.444444444
RELA	21	0.007877059	0.440677966
AKT1	19	0.005786231	0.43697479
TNF	18	0.008563792	0.435146444
CASP3	16	0.003892968	0.43153527
CCND1	16	0.004433613	0.43153527
IL-6	16	0.004364471	0.43153527
JUN	16	0.004582839	0.43153527
MMP9	16	0.006279692	0.43153527
NOS2	16	0.010049384	0.427983539
TP53	16	0.004451	0.43153527

**Table 6:** Characteristic parameters of target nodes of main active components of Danhong injection (Top 10).

Targets	Degree	Betweenness Centrality	Closeness centrality
PD-L1 expression and PD-1 checkpoint pathway in cancer	92	0.146538435	0.602898551
Lipid and atherosclerosis	84	0.10778941	0.563685637
Proteoglycans in cancer	81	0.119918588	0.566757493
Pathways in cancer	65	0.063874845	0.513580247
PI3K-Akt signaling pathway	50	0.019555913	0.385899814
AGE-RAGE signaling pathway in diabetic complications	45	0.015647294	0.380255941
NF-kappa B signaling pathway	42	0.029205678	0.459161148
Small cell lung cancer	41	0.025336488	0.457142857
Malaria	29	0.006503325	0.34608985
HIF-1 signaling pathway	26	0.005352865	0.360485269

#### 3.2.6. Molecular docking verification

**Figure 9.**
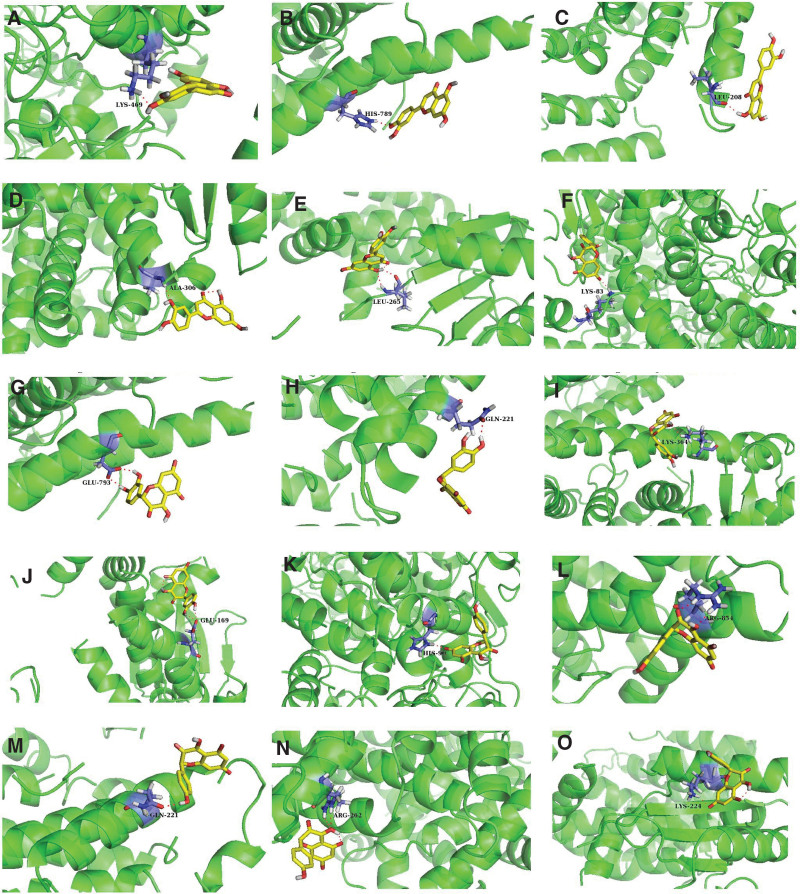
Molecular docking pattern diagram (AutoDock, version 4.2.6, Supported by The Scripps Research Institute). (A) Luteolin-PTGS2: they are docked at LYS-469, and the binding energy is −2.25 kJ/mol^−1^. (B) Luteolin-AR: they are docked at HIS-789, and the binding energy is −1.85 kJ/mol^−1^. (C) Luteolin-ESR1: they are docked at LEU-208, and the binding energy is −5.27 kJ/mol^−1^. (D) Luteolin-PPARG: they are docked at ALA-306, and the binding energy is −3.45 kJ/mol^−1^. (E) Luteolin-RELA: they are docked at LEU-265, and the binding energy is −3.09 kJ/mol^−1^. (F) Quercetin-PTGS2: they are docked at LYS-83, and the binding energy is −2.82 kJ/mol^−1^. (G) Quercetin-AR: they are docked at GLU-793, and the binding energy is −2.9 kJ/mol^−1^. (H) Quercetin-ESR1: they are docked at GLN-221, and the binding energy is −2.67 kJ/mol^−1^. (I) Quercetin-PPARG: they are docked at LYS-364, and the binding energy is −3.11 kJ/mol^−1^. (J) Quercetin-RELA: they are docked at GLU-169, and the binding energy is −2.91 kJ/mol^−1^. (K) Kaempferol-PTGS2: they are docked at HIS-90, and the binding energy is −2.31 kJ/mol^−1^. (L) Kaempferol-AR: they are docked at ARG-854, and the binding energy is −3.04 kJ/mol^−1^. (M) Kaempferol-ESR1: they are docked at GLN-221, and the binding energy is −2.96 kJ/mol^−1^. (N) Kaempferol-PPARG: they are docked at ARG-262, and the binding energy is −3.3 kJ/mol^−1^. (O) Kaempferol-RELA: they are docked at LYS-224, and the binding energy is −3.22 kJ/mol^−1^.

The top 3 core active ingredients (Luteolin, Quercetin, and Kaempferol) were molecularly docked with the core targets of IPF (PTGS2, AR, ESR1, PPARG, and RELA). The results showed that the binding energies of the core active ingredients to the targets were <0 (<0 kJ/mol^−1^), which indicated that the active ingredients in Danhong injection had good binding activity to the core targets of IPF. As shown in Table 7 and Figure 9.

**Table 7:** Binding energies of molecular docking of core compounds in Danhong injection.

ID	MOL ID	Compound	Molecular formula	Molecular weight (g/mol)	Target protein	Binding energy (kJ/mol^−1^)
DH3	MOL000006	Luteolin	C15H10O6	286.24	PTGS2	−2.25
					AR	−1.85
					ESR1	−5.27
					PPARG	−3.45
					RELA	−3.09
HH19	MOL000098	Quercetin	C15H10O7	302.23	PTGS2	−2.82
					AR	−2.9
					ESR1	−2.67
					PPARG	−3.11
					RELA	−2.91
HH16	MOL000422	Kaempferol	C15H10O6	286.24	PTGS2	−2.31
					AR	−3.04
					ESR1	−2.96
					PPARG	−3.3
					RELA	−3.22

DH = Danshen (*S miltiorrhiza* Bunge) and Honghua (Carthami Flos), HH = Honghua (Carthami Flos).

## 4. Discussion

In this study, the clinical efficacy and safety of Danhong injection in the treatment of IPF were evaluated by meta-analysis, and the targets and molecular mechanism of Danhong injection in the treatment of IPF were further explored by combining network pharmacology and molecular docking methods. The obtained results were satisfying. In meta-analysis, the results confirmed that Danhong injection, as an adjuvant treatment, performed better in improving clinical efficacy, lung function (FEV_1_/FVC%, FVC(L)), analysis of the arterial blood gas (DLCO, PaO_2_), inflammatory cytokines (TNF-α, TGF-β_1_), and serum cytokines associated with pulmonary fibrosis (HA, LN, PCIII, ColIII, BUN), which is suggesting that Danhong injection has certain advantages and curative effects as a alternative drug for the treatment of IPF. Regarding the safety of Danhong injection, there was only 1 description of adverse reactions in all the literatures, but the main cause was the side effects of the combination of medications, and the incidence of adverse reactions in patients was negligible. In addition, 2 other studies showed no adverse reactions. These results show that Danhong injection has a good safety profile. Some researchers used systems biology methods to simulate the mechanism of the compatibility of *S miltiorrhiza* Bunge and Carthami Flos (The 2 main Chinese medicines contained in Danhong injection) and found that they may play synergistic roles in the treatment of diseases mainly in biological pathways such as NF-κB cascade reaction, autophagy, proliferation, and migration.^[38]^ Some studies also showed that Danhong injection exerted a multi-target anti-inflammatory effect by inhibiting the expression of tumor necrosis factor-α (TNF-α) and other cytokines.^[39]^

However, the targets and molecular mechanism of Danhong injection in the treatment of IPF are still unclear. Therefore, we further explored the targets and molecular mechanism of Danhong injection in the treatment of IPF. In the network pharmacology, the targets of the main components of Danhong injection and the targets of IPF were screened and it was found that there were 221 related intersection target proteins between Danhong injection and IPF. Subsequently, PPI interaction analysis and the construction of the main components of Danhong injection-IPF main targets-pathways network were carried out, and the core components, core targets, and main signaling pathways of Danhong injection acting on IPF were obtained, and the molecular docking verification of the core components and core targets of Danhong injection acting on IPF was carried out.

Network pharmacological results showed that the core components of Danhong injection acting on IPF were mainly concentrated in Luteolin, Quercetin, and Kaempferol. Luteolin was a natural flavonoid that was widely found in many plants.^[40,41]^ Luteolin has a variety of biological effects, such as anti-inflammatory, antioxidant, and anticancer.^[42]^ Luteolin was found to improve collagen deposition effectively, transforming growth factor-β1 (TGF-β1) expression and pulmonary fibrosis in the lung tissues of mice induced by bleomycin (BLM), and similar results were found in vitro studies. The mechanism of these results was related to attenuation of TGF-β1-induced phosphorylation of Smad549. It is been shown that Luteolin can exert anti-pulmonary fibrosis effects by inhibiting lung inflammation and inhibiting myofibroblast differentiation and epithelial to mesenchymal transformation.^[43]^ In terms of anticancer, studies have shown that Luteolin hinder cancer progression through a variety of mechanisms, including inhibition of protein kinase, regulation of cell cycle, induction of apoptosis, induction of DNA damage, and reduction of transcription factors.^[44,45]^ In addition, in the model of lipopolysaccharide (LPS)-induced acute lung injury in mice, Luteolin suppressed the inflammatory response in lung tissue in a concentration-dependent manner and reduced the levels of TNF-α, IL-6, iNOS, and cyclooxygenase-2 (COX-2). The mechanism was related to the inhibition of the activation of NF-κB and its upstream molecular factor Akt.^[46]^ Luteolin can also prevent liver fibrosis by acting on the toll-like receptor (TLR) signaling pathway to reduce extracellular matrix accumulation and cathepsin gene expression while enhancing the liver’s antioxidant system.^[47]^ As a natural polyhydroxyflavonoid, Quercetin was widely present in nature. It has a variety of physiological activities such as oxidative stress, reducing inflammatory response, antitumor, cardiovascular protection, antihypertensive, antithrombotic, anti-adhesion, anti-atherosclerotic effects, etc.^[48–50]^ It also has intracellular antioxidant activity that inhibits the production of hydrogen peroxide-induced intracellular ROS in mouse fibroblasts, thereby mitigating diseases caused by oxidative stress.^[51]^ In the BLM-induced pulmonary fibrosis model of rats, it was found that the antioxidant capacity of rat lung tissue was significantly reduced, and the histopathological structure of lung was significantly improved after the intervention with Quercetin.^[52]^ In addition, Quercetin had anti-inflammatory effects in rats and patients with IPF, as evidenced by its ability to reduce damage caused by inflammatory cell infiltration and reduce the amount of inflammatory factors TNF-α and IL-8.^[53,54]^ It also inhibits the progression of PF by inhibiting the proliferation of mouse alveolar type II epithelial cells and NIH3T3 cells induced by TGF-β1 signaling, thereby reducing collagen and hyaluronic acid secretion^[55]^; it can improve PF by inhibiting the expression of TGF-β1-induced α-SMA, fibronectin, and collagen by affecting SphK1 or S1P3/SphK signaling pathways^[56,57]^; And it can upregulate the expression of FasL receptor and reverse the resistance of fibrotic cells to apoptosis, thereby alleviating BLM-induced PF in mice^[58]^; it also promotes the secretion of MMPs by human embryonic lung fibroblasts, restoring the balance of MMP-1/TIMP-1 to reduce lung fiber maintenance.^[59]^ Kaempferol was a natural flavonoid found widely in many fruits and vegetables^[60]^ with anti-inflammatory, antioxidant, and antibacterial effects.^[61]^ Kaempferol has been found to alleviate fibrotic airway remodeling by inactivating PAR1 in epithelial cells,^[62]^ and to protect mice from acute lung injury by regulating TRAF6 ubiquitination^[63]^ and by inhibiting the expression of oxidative stress proteins iNOS (NOS2) and ICAM-1.^[64]^ In addition, Kaempferol has shown significant antifibrotic effects in cardiac remodeling^[61]^ and it can also inhibit the progression of silica-induced PF.^[65]^ Therefore, the core active ingredients of Danhong injection (Luteolin, Quercetin, and Kaempferol) mainly improve IPF by anti-inflammatory, oxidative stress, induction of apoptosis, inhibition of cell proliferation, regulation of immunity, induction of DNA damage, reduction of transcription factors, etc.

Network pharmacological results showed that the main targets of Danhong injection on IPF were PTGS2, AR, ESR1, PPARG, RELA, etc. Prostaglandin-endoperoxide synthase 2 (PTGS2) is the gene encoding cyclooxygenase-2 (COX-2), which is an inducible enzyme that can participate in various pathological processes such as inflammation, cell proliferation, and apoptosis in various cells.^[66]^ It is been reported that COX-2 was highly expressed in bleomycin-induced pulmonary fibrosis tissues.^[67]^ Androgens can increase the androgen receptor (AR) protein levels in lung cells, and AR was mainly expressed in alveolar epithelial cells (AECs) II and bronchial epithelial cells of mouse lungs. In lung cancer studies, TMPRSS2 has been found to be one of the genes upregulated by androgen exposure in A549 cells and is a direct target of AR.^[68]^ Estrogen (E2) (17β-estradiol) can activate several receptors, including the nuclear transcription factor estrogen receptor α (ESR1) and estrogen receptor β (ESR2). ESR1 expression has been found to be higher than ESR2 in human lung adenocarcinoma (LUAD) and lung squamous cell carcinoma (LUSC), suggesting that ESR1 may play a major role in inducing lung cancer (LC) cells.^[69]^ However, in another study, exposure to TGF-β1 in bronchial epithelial cells resulted in dose-dependent and significantly reduced ESR1 and ESR2 mRNA and protein expression.^[70]^ Therefore, whether ESR1 has a protective or inducing effect on pulmonary fibrosis is controversial. PPARG encoded peroxisome proliferator-activated receptor γ (PPARγ), and the antifibrotic effect of PPARγ has been found to be manifested by inhibiting TGF-β expression and blocking downstream signal transduction after ligand activation.^[71]^ Furthermore, Metformin induced adipose differentiation in myofibroblasts by upregulating BMP2 and activating PPARγ.^[72]^ These results indicate the progression of negative regulatory fibrosis by activation of PPARγ and suggest that this receptor may be a key target for the treatment of IPF. The NF-κB signaling pathway is involved in inflammatory response, cell proliferation, cell differentiation, cell adhesion and growth signaling, apoptosis defense, and host immune response.^[73]^ There are many basic transcription factors in the NF-κB signaling pathway, among which the gene encoding transcription factor p65 is RELA.^[74]^ Studies have shown that the inhibition of autophagy in alveolar epithelial cells of IPF patients is accompanied by p65/RELA-mediated activation of SNAI2, a process that not only controls epithelial–mesenchymal transition (EMT), but also regulates the production of locally acting profibrotic mediators.^[75]^ In addition, NF-κB/RelA was found to be the major mediator of TGF-β-induced EMT in vitro models of telomerase immortalized epithelial cells^[76]^: TGF-β activated NF-κB/RelA and induced its nuclear translocation, and NF-κB/RelA can directly upregulate multiple core EMT regulators in the nucleus.^[77]^ Therefore, the main functions of PTGS2, AR, ESR1, PPARG, and RELA in the formation of IPF can be summarized as: pro-inflammatory response, regulation of cell adhesion and growth signals, control of cell proliferation, differentiation and apoptosis, regulation of oxidative stress response, and regulation of host immune response. These functions are consistent with the effect of the core active ingredients (Luteolin, Quercetin, and Kaempferol) of the aforementioned Danhong injection in improving IPF, and the results of molecular docking verification also show that Luteolin, Quercetin, and Kaempferol have strong binding activity to these targets. Therefore, it can be considered that Danhong injection mainly acts on PTGS2, AR, ESR1, PPARG, RELA, and other targets to treat IPF.

In network pharmacology, KEGG pathway analysis found that Danhong injection was mainly through PD-L1 expression and PD-1 checkpoint pathway in cancer (PPCPC), LA, proteoglycans in cancer (PRC), PC, PASP, etc to treat IPF. Studies showed that although the cell origin and pathological phenotype were different, the pathogenesis of IPF and LC was highly similar, they both have chronic damage to AECs accompanied by abnormal tissue repair and alveolar structure damage.^[78,79]^ Therefore, it can be considered that the mechanism of IPF and cancer production is similar, and related cytokines can act on PC and PRC to induce IPF production. Programmed death receptor 1 (PD-1) on T cells is involved in regulating T cell function under normal physiological conditions.^[80]^ After the activation of effector T cells, the programmed death ligand 1 (PD-L1) of PD-1 is upregulated in peripheral tissues.^[80,81]^ Subsequent binding of PD-1 to PD-L1 results in inhibitory co-stimulation signaling of T cell receptors (TCRs), a process that is essential for maintaining immune tolerance and preventing autoimmune pathology.^[82]^ Studies have found that PD-L1 expression is present in lung fibrosis-related tumor cells of 62% of patients and the PD-1/PD-L1 axis is induced. These results confirmed the potential role of PD-1/PD-L1 checkpoint in IPF.^[83]^ Moreover, serum expression of soluble PD-L1 had increased threefold in patients with IPF.^[84]^ In addition, other studies found that the expression of PD-L1 in peripheral blood of IPF patients did not increase, but the expression of PD-1 in T lymphocytes in peripheral blood and lung tissue of IPF patients increased significantly.^[85]^ These studies suggest that PD-1/PD-L1 checkpoints are induced in IPF regardless of the actual cell type expressing PD-1 or PD-L1, therefore immune editing may affect the disease progression of IPF. Most vascular resistance in pulmonary fibrosis is due to persistent vasoconstriction,^[86]^ which has some common ground with pulmonary hypertension (PAH). Many of the pathophysiological mechanisms found in PAH overlap with those associated with atherosclerosis, including vascular smooth muscle and endothelial cell dysfunction.^[87]^ Furthermore, prostacyclin (PGI 2) has been found to play a central role in the treatment of PAH through its powerful vasodilating, antithrombotic, and antiproliferative effects.^[88]^ Therefore, the pathological process of IPF is also related to the mechanism of atherosclerosis. The PI3K/Akt/mTOR signaling pathway is one of the important signal transduction pathways in cells, which plays an important role in regulating physiological functions such as cell growth, proliferation, motility, cell survival, autophagy, protein synthesis, and transcription.^[89]^ In PI3K/Akt/mTOR, phosphoesterinositol-3-kinase (PI3K) is a signal transductase that is the initiation factor of the PI3K/Akt/mTOR pathway.^[90]^ Moreover, protein kinase B (PKB: alias Akt) is located in downstream of PI3K, and it can activate downstream mTOR and others to participate in cell differentiation and proliferation^[91,92]^ when it is phosphorylated. Studies has shown that it plays a key regulatory role in the formation of IPF: PI3K/Akt/mTOR is highly expressed in AECs II in the IPF rat model, thereby inhibiting autophagy, promoting collagen synthesis, and IPF.^[93]^ Therefore, these signaling pathways mainly play the role of pro-inflammatory response, regulation of oxidative stress response, regulation of immune response, control of apoptosis, cell proliferation, and regulation of vascular endothelial cell function in the formation of IPF.

Combined with the above results, it can be considered that Danhong injection is mainly expressed through PPCPC, LA, and PRC, PC, PASP act on PTGS2, AR, ESR1, PPARG, RELA, and other targets to treat IPF.

Limitations of the study: meta-analysis: all 12 literatures only mentioned “random,” but did not indicate a specific method of randomization. In addition, no literature reported on allocation concealment and blinding. Therefore, the literature included in this meta-analysis may be biased; these studies were unicentre and had small sample sizes. In addition, they have some clinical heterogeneity, such as a different course of treatment and a different course of disease. These reasons may reduce the reliability of this meta-analysis; all the studies had no report of relevant death and endpoint indicators, and no description of the patient’s follow-up, so it is nearly impossible to reach the conclusion that whether Danhong injection can better reduce the patient’s mortality and other endpoint events or not. Network pharmacology: based on the results of bioinformatics and massive data calculation, this study preliminarily elucidated the mechanism of the action of Danhong injection in the treatment of IPF from the perspective of network pharmacology. However, since most of the databases, such as GeneCards, derive their data on disease-associated genes from results reported in a portion of the previous literature, their respective data may not be particularly complete. Therefore, there may be inconsistencies between the results of these databases and those reported in the few available literature. These results should be subsequently validated by in vivo or in vitro experiments to further clarify the main targets and molecular mechanisms of Danhong injection in regulating IPF.

## 5. Conclusion

As an adjuvant treatment, Danhong injection can alleviate the clinical symptoms of IPF patients, increase lung function, improve arterial blood gas analysis, and reduce inflammatory factors and serum cytokine levels, therefore, it is truly effective. The core active components of Danhong Injection (Luteolin, Quercetin, and Kaempferol) mainly act on targets such as PTGS2, AR, ESR1, PPARG, and RELA through signal transduction of PPCPC, LA, PRC, PC, and PASP to play the role of anti-inflammatory, anti-oxidative stress, inducing apoptosis, inhibiting cell proliferation, regulating immunity, inducing DNA damage and reducing transcription factors to reduce fibronectin and collagen, inhibit extracellular matrix deposition and airway remodeling to improve IPF. This conclusion provides a scientific basis for the clinical use of Danhong injection for the treatment of IPF and provides a new direction to explore the potential mechanism of action of Danhong injection.

## Author contributions

**Conceptualization:** Xiaozheng Wu.

**Data curation:** Xiaozheng Wu.

**Formal analysis:** Xiaozheng Wu.

**Funding acquisition:** Xiaozheng Wu.

**Investigation:** Xiaozheng Wu.

**Methodology:** Xiaozheng Wu, Wen Li.

**Project administration:** Xiaozheng Wu.

**Resources:** Xiaozheng Wu.

**Software:** Xiaozheng Wu.

**Validation:** Xiaozheng Wu.

**Visualization:** Xiaozheng Wu.

**Writing – original draft:** Xiaozheng Wu.

**Writing – review & editing:** Wen Li, Zhenliang Luo, Yunzhi Chen.

**Supervision:** Yunzhi Chen.

## Supplementary Material
















































