# Supplementary material for: Exploring the efficacy and molecular mechanism of Danhong injection comprehensively in the treatment of idiopathic pulmonary fibrosis by combining meta-analysis, network pharmacology, and molecular docking methods
Source: Medicine (Baltimore). 2024 May 10;103(19):e38133. doi: 10.1097/MD.0000000000038133 (PMC11081554; doi:10.1097/MD.0000000000038133)
Supplement: Supplementary file 1 [file medi-103-e38133-s001.docx]

**Table S1 PubMed search strategy**

| **Number** | **Search Terms** |
| --- | --- |
| **#1** | Mesh descriptor: (Idiopathic pulmonary fibrosis) explode all trees |
| **#2** | ((((((Pulmonary fibrosisor[Title/Abstract]) OR Pulmonary interstitial fibrosis [Title/Abstract]) OR Interstitial lung disease [Title/Abstract]) OR IPF [Title/Abstract]) |
| **#3** | Or 1-2 |
| **#4** | Mesh descriptor: (Danhong) explode all trees |
| **#5** | ((((((Danhong injection[Title/Abstract]) OR Danhong[Title/Abstract]) |
| **#6** | Or 4-5 |
| **#7** | RCT[Title/Abstract]) OR Randomized controlled trial[Title/Abstract]) |
| **#8** | 3 and 6 and 7 |
