# Supplementary material for: Exploring the efficacy and molecular mechanism of Danhong injection comprehensively in the treatment of idiopathic pulmonary fibrosis by combining meta-analysis, network pharmacology, and molecular docking methods
Source: Medicine (Baltimore). 2024 May 10;103(19):e38133. doi: 10.1097/MD.0000000000038133 (PMC11081554; doi:10.1097/MD.0000000000038133)
Supplement: Supplementary file 2 [file medi-103-e38133-s002.docx]

**Table S2 Quality evaluation of studies**

| Studies | Randomization method | Allocation concealment | Blind method | Loss to follow-up | Baseline comparability | Jadad score |
| --- | --- | --- | --- | --- | --- | --- |
| Cai 2015[24] | Random | Not described | Not described | Adverse reactions | No significant difference | 3 |
| Chen 2014[25] | Random number table method | Not described | Not described | Not described | No significant difference | 3 |
| Li 2012[26] | Random | Not described | Not described | Not described | No significant difference | 2 |
| Lin 2016[27] | Random | Not described | Not described | Not described | No significant difference | 2 |
| Ren 2012[28] | Random | Not described | Not described | Not described | No significant difference | 2 |
| Sun 2015[29] | Random number table method | Not described | Not described | Not described | No significant difference | 3 |
| Wang 2016[30] | Random | Not described | Not described | Not described | No significant difference | 2 |
| Wang 2020[31] | Random | Not described | Not described | Not described | No significant difference | 2 |
| Wu 2018[32] | Random number table method | Not described | Not described | Not described | No significant difference | 3 |
| Yin 2011[33] | Random | Not described | Not described | Not described | No significant difference | 2 |
| Zhao 2016[34] | Random | Not described | Not described | Not described | No significant difference | 2 |
| Zhou 2012[35] | Random | Not described | Not described | Not described | No significant difference | 2 |
