# Supplementary figures and images for: Exploring the efficacy and molecular mechanism of Danhong injection comprehensively in the treatment of idiopathic pulmonary fibrosis by combining meta-analysis, network pharmacology, and molecular docking methods
Source: Medicine (Baltimore). 2024 May 10;103(19):e38133. doi: 10.1097/MD.0000000000038133 (PMC11081554; doi:10.1097/MD.0000000000038133)

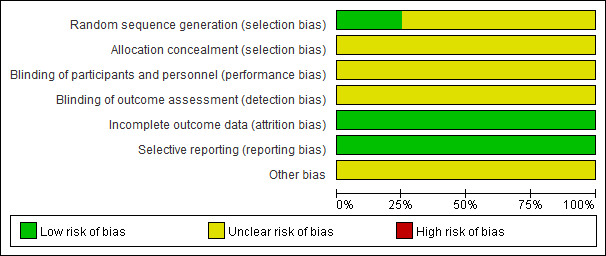

Supplement: Supplementary file 3 [file medi-103-e38133-s003.doc]

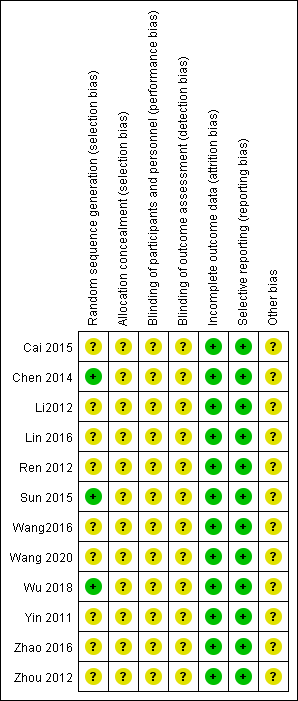

Supplement: Supplementary file 4 [file medi-103-e38133-s004.doc]

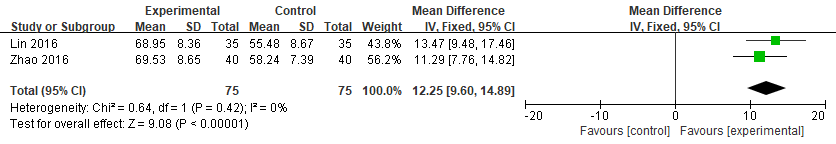

Supplement: Supplementary file 5 [file medi-103-e38133-s005.doc]

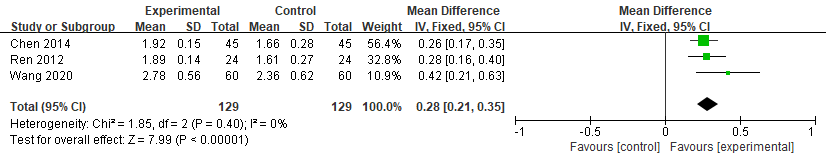

Supplement: Supplementary file 6 [file medi-103-e38133-s006.doc]

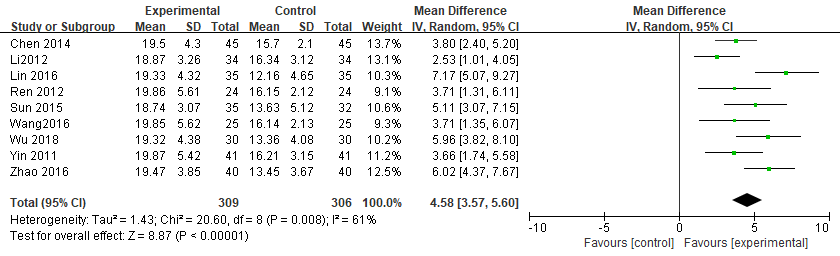

Supplement: Supplementary file 7 [file medi-103-e38133-s007.doc]

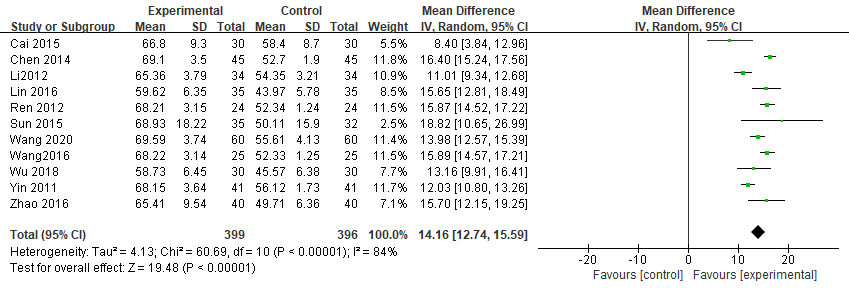

Supplement: Supplementary file 8 [file medi-103-e38133-s008.doc]

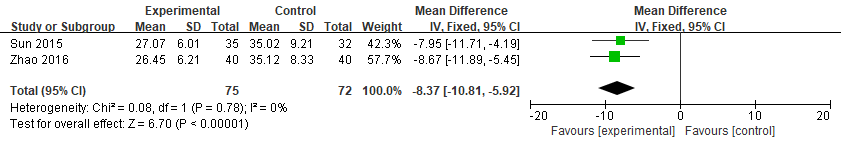

Supplement: Supplementary file 9 [file medi-103-e38133-s009.doc]

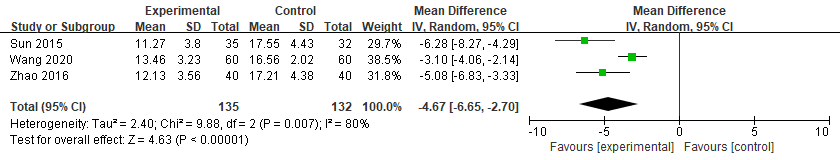

Supplement: Supplementary file 10 [file medi-103-e38133-s010.doc]
