# Supplementary material for: Exploring the efficacy and molecular mechanism of Danhong injection comprehensively in the treatment of idiopathic pulmonary fibrosis by combining meta-analysis, network pharmacology, and molecular docking methods
Source: Medicine (Baltimore). 2024 May 10;103(19):e38133. doi: 10.1097/MD.0000000000038133 (PMC11081554; doi:10.1097/MD.0000000000038133)
Supplement: Supplementary file 16 [file medi-103-e38133-s016.docx]

**Table S3 The results of bias by Begg's Test (Clinical efficacy)**

| Begg's Test | |
| --- | --- |
| adj. Kendall's Score (P-Q) | 1 |
| Std. Dev. of Score | 5.32 |
| Number of Studies | 6 |
| z | 0.19 |
| Pr > \|z\| | 0.851 |
| z | 0.00 (continuity corrected) |
| Pr > \|z\| | 1.000 (continuity corrected) |
