# Supplementary material for: Exploring the efficacy and molecular mechanism of Danhong injection comprehensively in the treatment of idiopathic pulmonary fibrosis by combining meta-analysis, network pharmacology, and molecular docking methods
Source: Medicine (Baltimore). 2024 May 10;103(19):e38133. doi: 10.1097/MD.0000000000038133 (PMC11081554; doi:10.1097/MD.0000000000038133)
Supplement: Supplementary file 17 [file medi-103-e38133-s017.docx]

**Table S4 The results of bias by Egger's test (Clinical efficacy)**

| Egger's test | | | | | | |
| --- | --- | --- | --- | --- | --- | --- |
| Std_Eff | Coef. | Std. Err. | t | P>\|t\| | [95% Conf. Interval] | |
| slope | 0.6548491 | 0.6143614 | 1.07 | 0.347 | -1.050892 | 2.36059 |
| bias | 1.126738 | 0.9423969 | 1.20 | 0.298 | -1.489775 | 3.743251 |
