# Supplementary material for: Exploring the efficacy and molecular mechanism of Danhong injection comprehensively in the treatment of idiopathic pulmonary fibrosis by combining meta-analysis, network pharmacology, and molecular docking methods
Source: Medicine (Baltimore). 2024 May 10;103(19):e38133. doi: 10.1097/MD.0000000000038133 (PMC11081554; doi:10.1097/MD.0000000000038133)
Supplement: Supplementary file 20 [file medi-103-e38133-s020.docx]

**Table S5 Influence analysis results data of clinical efficacy**

| Study omitted | Estimate | [95% Conf. Interval] | |
| --- | --- | --- | --- |
| Li (2012) | 3.9401515 | 2.2336936 | 6.9502788 |
| Ren (2012) | 4.2391024 | 2.4423537 | 7.3576517 |
| Sun (2015) | 3.6985137 | 2.1623335 | 6.3260384 |
| Wang (2016) | 4.2708297 | 2.4377053 | 7.4824414 |
| Yin (2011) | 4.1515303 | 2.3001008 | 7.4932384 |
| Zhou (2012) | 4.0703659 | 2.238739 | 7.4005404 |
| Combined | 4.0525105 | 2.4169736 | 6.7947953 |
