# Supplementary material for: Exploring the efficacy and molecular mechanism of Danhong injection comprehensively in the treatment of idiopathic pulmonary fibrosis by combining meta-analysis, network pharmacology, and molecular docking methods
Source: Medicine (Baltimore). 2024 May 10;103(19):e38133. doi: 10.1097/MD.0000000000038133 (PMC11081554; doi:10.1097/MD.0000000000038133)
Supplement: Supplementary file 22 [file medi-103-e38133-s022.docx]

**Table S6 Main components of Danhong injection**

| Drug | ID | MOL ID | Molecule Name | OB(%) | DL | Molecular Formula |
| --- | --- | --- | --- | --- | --- | --- |
| Salvia miltiorrhiza Bunge (Danshen) | DS1 | MOL001601 | 1,2,5,6-tetrahydrotanshinone | 38.75 | 0.36 | C18H16O3 |
|  | DS2 | MOL001659 | Poriferasterol | 43.83 | 0.76 | C29H48O |
|  | DH1 | MOL001771 | poriferast-5-en-3beta-ol | 36.91 | 0.75 | C29H50O |
|  | DS3 | MOL001942 | isoimperatorin | 45.46 | 0.23 | C16H14O4 |
|  | DS4 | MOL002222 | sugiol | 36.11 | 0.28 | C20H28O2 |
|  | DS5 | MOL002651 | Dehydrotanshinone II A | 43.76 | 0.4 | C19H16O3 |
|  | DH2 | MOL002776 | Baicalin | 40.12 | 0.75 | C21H18O11 |
|  | DS6 | MOL000569 | digallate | 61.85 | 0.26 | [C14H10O9](https://pubchem.ncbi.nlm.nih.gov/#query=C14H10O9) |
|  | DH3 | MOL000006 | luteolin | 36.16 | 0.25 | C15H10O6 |
|  | DS7 | MOL006824 | α-amyrin | 39.51 | 0.76 | C30H50O |
|  | DS8 | MOL007036 | 5,6-dihydroxy-7-isopropyl-1,1-dimethyl-2,3-dihydrophenanthren-4-one | 33.77 | 0.29 | [C19H22O3](https://pubchem.ncbi.nlm.nih.gov/#query=C19H22O3) |
|  | DS9 | MOL007041 | 2-isopropyl-8-methylphenanthrene-3,4-dione | 40.86 | 0.23 | [C18H16O2](https://pubchem.ncbi.nlm.nih.gov/#query=C18H16O2) |
|  | DS10 | MOL007045 | 3α-hydroxytanshinoneⅡa | 44.93 | 0.44 | C19H18O4 |
|  | DS11 | MOL007048 | (E)-3-[2-(3,4-dihydroxyphenyl)-7-hydroxy-benzofuran-4-yl]acrylic acid | 48.24 | 0.31 | [C17H12O6](https://pubchem.ncbi.nlm.nih.gov/#query=C17H12O6) |
|  | DS12 | MOL007049 | 4-methylenemiltirone | 34.35 | 0.23 | [C18H18O2](https://pubchem.ncbi.nlm.nih.gov/#query=C18H18O2) |
|  | DS13 | MOL007050 | 2-(4-hydroxy-3-methoxyphenyl)-5-(3-hydroxypropyl)-7-methoxy-3-benzofurancarboxaldehyde | 62.78 | 0.4 | C20H20O6 |
|  | DS14 | MOL007051 | 6-o-syringyl-8-o-acetyl shanzhiside methyl ester | 46.69 | 0.71 | C28H36O16 |
|  | DS15 | MOL007058 | formyltanshinone | 73.44 | 0.42 | [C18H10O4](https://pubchem.ncbi.nlm.nih.gov/#query=C18H10O4) |
|  | DS16 | MOL007059 | 3-beta-Hydroxymethyllenetanshiquinone | 32.16 | 0.41 | [C18H14O4](https://pubchem.ncbi.nlm.nih.gov/#query=C18H14O4) |
|  | DS17 | MOL007061 | Methylenetanshinquinone | 37.07 | 0.36 | [C18H14O3](https://pubchem.ncbi.nlm.nih.gov/#query=C18H14O3) |
|  | DS18 | MOL007063 | przewalskin a | 37.11 | 0.65 | C23H26O6 |
|  | DS19 | MOL007064 | przewalskin b | 110.32 | 0.44 | C20H26O4 |
|  | DS20 | MOL007068 | Przewaquinone B | 62.24 | 0.41 | [C18H12O4](https://pubchem.ncbi.nlm.nih.gov/#query=C18H12O4) |
|  | DS21 | MOL007069 | przewaquinone c | 55.74 | 0.4 | C18H16O4 |
|  | DS22 | MOL007070 | (6S,7R)-6,7-dihydroxy-1,6-dimethyl-8,9-dihydro-7H-naphtho[8,7-g]benzofuran-10,11-dione | 41.31 | 0.45 | C18H16O5 |
|  | DS23 | MOL007071 | przewaquinone f | 40.31 | 0.46 | C18H16O5 |
|  | DS24 | MOL007077 | sclareol | 43.67 | 0.21 | [C20H36O2](https://pubchem.ncbi.nlm.nih.gov/#query=C20H36O2) |
|  | DS25 | MOL007079 | tanshinaldehyde | 52.47 | 0.45 | C19H16O4 |
|  | DS26 | MOL007081 | Danshenol B | 57.95 | 0.56 | [C22H26O4](https://pubchem.ncbi.nlm.nih.gov/#query=C22H26O4) |
|  | DS27 | MOL007082 | Danshenol A | 56.97 | 0.52 | [C21H20O4](https://pubchem.ncbi.nlm.nih.gov/#query=C21H20O4) |
|  | DS28 | MOL007085 | Salvilenone | 30.38 | 0.38 | C20H20O2 |
|  | DS29 | MOL007088 | cryptotanshinone | 52.34 | 0.4 | [C19H20O3](https://pubchem.ncbi.nlm.nih.gov/#query=C19H20O3) |
|  | DS30 | MOL007093 | dan-shexinkum d | 38.88 | 0.55 | C21H20O4 |
|  | DS31 | MOL007094 | danshenspiroketallactone | 50.43 | 0.31 | C17H16O3 |
|  | DS32 | MOL007098 | deoxyneocryptotanshinone | 49.4 | 0.29 | C19H22O3 |
|  | DS33 | MOL007100 | dihydrotanshinlactone | 38.68 | 0.32 | C17H14O3 |
|  | DS34 | MOL007101 | dihydrotanshinoneⅠ | 45.04 | 0.36 | C18H14O3 |
|  | DS35 | MOL007105 | epidanshenspiroketallactone | 68.27 | 0.31 | C17H16O3 |
|  | DS36 | MOL007107 | C09092 | 36.07 | 0.25 | C20H30O |
|  | DS37 | MOL007108 | isocryptotanshi-none | 54.98 | 0.39 | C19H20O3 |
|  | DS38 | MOL007111 | Isotanshinone II | 49.92 | 0.4 | [C19H18O3](https://pubchem.ncbi.nlm.nih.gov/#query=C19H18O3) |
|  | DS39 | MOL007115 | manool | 45.04 | 0.2 | C21H36O |
|  | DS40 | MOL007118 | microstegiol | 39.61 | 0.28 | C20H26O2 |
|  | DS41 | MOL007119 | miltiononeⅠ | 49.68 | 0.32 | [C19H20O4](https://pubchem.ncbi.nlm.nih.gov/#query=C19H20O4) |
|  | DS42 | MOL007120 | miltiononeⅡ | 71.03 | 0.44 | [C19H20O4](https://pubchem.ncbi.nlm.nih.gov/#query=C19H20O4) |
|  | DS43 | MOL007121 | miltipolone | 36.56 | 0.37 | C19H24O3 |
|  | DS44 | MOL007122 | Miltirone | 38.76 | 0.25 | [C19H22O2](https://pubchem.ncbi.nlm.nih.gov/#query=C19H22O2) |
|  | DS45 | MOL007123 | miltironeⅡ | 44.95 | 0.24 | C16H16O4 |
|  | DS46 | MOL007124 | neocryptotanshinone ii | 39.46 | 0.23 | [C17H18O3](https://pubchem.ncbi.nlm.nih.gov/#query=C17H18O3) |
|  | DS47 | MOL007125 | neocryptotanshinone | 52.49 | 0.32 | [C19H22O4](https://pubchem.ncbi.nlm.nih.gov/#query=C19H22O4) |
|  | DS48 | MOL007127 | 1-methyl-8,9-dihydro-7H-naphtho[5,6-g]benzofuran-6,10,11-trione | 34.72 | 0.37 | [C17H12O4](https://pubchem.ncbi.nlm.nih.gov/#query=C17H12O4) |
|  | DS49 | MOL007130 | prolithospermic acid | 64.37 | 0.31 | C17H14O6 |
|  | DS50 | MOL007132 | (2R)-3-(3,4-dihydroxyphenyl)-2-[(Z)-3-(3,4-dihydroxyphenyl)acryloyl]oxy-propionic acid | 109.38 | 0.35 | [C18H16O8](https://pubchem.ncbi.nlm.nih.gov/#query=C18H16O8) |
|  | DS51 | MOL007140 | (Z)-3-[2-[(E)-2-(3,4-dihydroxyphenyl)vinyl]-3,4-dihydroxy-phenyl]acrylic acid | 88.54 | 0.26 | [C17H14O6](https://pubchem.ncbi.nlm.nih.gov/#query=C17H14O6) |
|  | DS52 | MOL007141 | salvianolic acid g | 45.56 | 0.61 | C18H12O7 |
|  | DS53 | MOL007142 | salvianolic acid j | 43.38 | 0.72 | C27H22O12 |
|  | DS54 | MOL007143 | salvilenoneⅠ | 32.43 | 0.23 | C20H20O2 |
|  | DS55 | MOL007145 | salviolone | 31.72 | 0.24 | [C18H20O2](https://pubchem.ncbi.nlm.nih.gov/#query=C18H20O2) |
|  | DS56 | MOL007149 | NSC 122421 | 34.49 | 0.28 | [C20H28O2](https://pubchem.ncbi.nlm.nih.gov/#query=C20H28O2) |
|  | DS57 | MOL007150 | (6S)-6-hydroxy-1-methyl-6-methylol-8,9-dihydro-7H-naphtho[8,7-g]benzofuran-10,11-quinone | 75.39 | 0.46 | [C18H16O5](https://pubchem.ncbi.nlm.nih.gov/#query=C18H16O5) |
|  | DS58 | MOL007151 | Tanshindiol B | 42.67 | 0.45 | [C18H16O5](https://pubchem.ncbi.nlm.nih.gov/#query=C18H16O5) |
|  | DS59 | MOL007152 | Przewaquinone E | 42.85 | 0.45 | [C18H16O5](https://pubchem.ncbi.nlm.nih.gov/#query=C18H16O5) |
|  | DS60 | MOL007154 | tanshinone iia | 49.89 | 0.4 | C19H18O3 |
|  | DS61 | MOL007155 | (6S)-6-(hydroxymethyl)-1,6-dimethyl-8,9-dihydro-7H-naphtho[8,7-g]benzofuran-10,11-dione | 65.26 | 0.45 | C19H18O4 |
|  | DS62 | MOL007156 | Tanshinone Ⅵ | 45.64 | 0.3 | C18H16O4 |
| Carthami  Flos (Honghua) | DH1 | MOL001771 | poriferast-5-en-3beta-ol | 36.91 | 0.75 | C29H50O |
|  | HH1 | MOL002680 | Flavoxanthin | 60.41 | 0.56 | [C40H56O3](https://pubchem.ncbi.nlm.nih.gov/#query=C40H56O3) |
|  | HH2 | MOL002694 | 4-[(E)-4-(3,5-dimethoxy-4-oxo-1-cyclohexa-2,5-dienylidene)but-2-enylidene]-2,6-dimethoxycyclohexa-2,5-dien-1-one | 48.47 | 0.36 | [C20H20O6](https://pubchem.ncbi.nlm.nih.gov/#query=C20H20O6) |
|  | HH3 | MOL002695 | lignan | 43.32 | 0.65 | C25H30O8 |
|  | HH4 | MOL002698 | lupeol-palmitate | 33.98 | 0.32 | C46H80O2 |
|  | HH5 | MOL002706 | Phytoene | 39.56 | 0.5 | [C40H64](https://pubchem.ncbi.nlm.nih.gov/#query=C40H64) |
|  | HH6 | MOL002707 | phytofluene | 43.18 | 0.5 | C40H62 |
|  | HH7 | MOL002710 | Pyrethrin II | 48.36 | 0.35 | [C22H28O5](https://pubchem.ncbi.nlm.nih.gov/#query=C22H28O5) |
|  | HH8 | MOL002712 | 6-Hydroxykaempferol | 62.13 | 0.27 | C15H10O7 |
|  | HH9 | MOL002714 | baicalein | 33.52 | 0.21 | C15H10O5 |
|  | HH10 | MOL002717 | qt_carthamone | 51.03 | 0.2 | C15H10O6 |
|  | HH11 | MOL002719 | 6-Hydroxynaringenin | 33.23 | 0.24 | [C15H12O6](https://pubchem.ncbi.nlm.nih.gov/#query=C15H12O6) |
|  | HH12 | MOL002721 | quercetagetin | 45.01 | 0.31 | C15H10O8 |
|  | HH13 | MOL002757 | 7,8-dimethyl-1H-pyrimido[5,6-g]quinoxaline-2,4-dione | 45.75 | 0.19 | [C12H10N4O2](https://pubchem.ncbi.nlm.nih.gov/#query=C12H10N4O2) |
|  | HH14 | MOL002773 | beta-carotene | 37.18 | 0.58 | [C40H56](https://pubchem.ncbi.nlm.nih.gov/#query=C40H56) |
|  | DH2 | MOL002776 | Baicalin | 40.12 | 0.75 | [C21H18O11](https://pubchem.ncbi.nlm.nih.gov/#query=C21H18O11) |
|  | HH15 | MOL000358 | beta-sitosterol | 36.91 | 0.75 | [C29H50O](https://pubchem.ncbi.nlm.nih.gov/#query=C29H50O) |
|  | HH16 | MOL000422 | kaempferol | 41.88 | 0.24 | [C15H10O6](https://pubchem.ncbi.nlm.nih.gov/#query=C15H10O6) |
|  | HH17 | MOL000449 | Stigmasterol | 43.83 | 0.76 | [C29H48O](https://pubchem.ncbi.nlm.nih.gov/#query=C29H48O) |
|  | DH3 | MOL000006 | luteolin | 36.16 | 0.25 | [C15H10O6](https://pubchem.ncbi.nlm.nih.gov/#query=C15H10O6) |
|  | HH18 | MOL000953 | CLR | 37.87 | 0.68 | C27H46O |
|  | HH19 | MOL000098 | quercetin | 46.43 | 0.28 | C15H10O7 |

Note: DH: Danshen (Salvia miltiorrhiza Bunge) and Honghua (Carthami Flos); HH: Honghua (Carthami Flos); DS: Danshen (Salvia miltiorrhiza Bunge).
