# Supplementary material for: Exploring the efficacy and molecular mechanism of Danhong injection comprehensively in the treatment of idiopathic pulmonary fibrosis by combining meta-analysis, network pharmacology, and molecular docking methods
Source: Medicine (Baltimore). 2024 May 10;103(19):e38133. doi: 10.1097/MD.0000000000038133 (PMC11081554; doi:10.1097/MD.0000000000038133)
Supplement: Supplementary file 23 [file medi-103-e38133-s023.docx]

**Table S7 Screening results of IPF-related targets**

| Items | Targets（n） |
| --- | --- |
| OMIM-Entry-Retrieval | 26 |
| OMIM-Gene-Map-Retrieval | 338 |
| TTD | 20 |
| DRUGBANK | 42 |
| disgenet | 804 |
| Genecards | 3054 |
| Merge and remove duplicate values | 1870 |
