# Supplementary material for: Exploring the efficacy and molecular mechanism of Danhong injection comprehensively in the treatment of idiopathic pulmonary fibrosis by combining meta-analysis, network pharmacology, and molecular docking methods
Source: Medicine (Baltimore). 2024 May 10;103(19):e38133. doi: 10.1097/MD.0000000000038133 (PMC11081554; doi:10.1097/MD.0000000000038133)
Supplement: Supplementary file 24 [file medi-103-e38133-s024.docx]

**Table S8 Enrichment results of main signal pathways of Danhong injection acting on IPF**

| GO | Description | Count | Log10(P) | Hits |
| --- | --- | --- | --- | --- |
| hsa05200 | Pathways in cancer | 65 | -83.42 | AKT1,XIAP,AR,CCND1,BCL2L1,CASP3,CASP8,CASP9,CCNA2,CDK4,CDKN1A,CDKN2A,CHUK,CTNNB1,EDN1,EGF,EGFR,EP300,ERBB2,ESR1,ESR2,FGFR1,FOS,HIF1A,HMOX1,HRAS,HSP90AA1,IFNG,IGF1R,IGF2,IKBKB,IL2,IL4,IL6,CXCL8,ITGB1,JAK2,JUN,KIT,MDM2,MET,MMP1,MMP2,MMP9,MYC,NFE2L2,NFKBIA,NOS2,PIK3CA,PPARG,MAPK1,MAPK3,MAPK8,PTEN,PTGS2,RAF1,RB1,RELA,STAT1,STAT3,TERT,TGFB1,TP53,VEGFA,CYCS |
| hsa05417 | Lipid and atherosclerosis | 43 | -62.76 | AKT1,BCL2L1,CASP1,CASP3,CASP8,CASP9,CD40LG,CHUK,MAPK14,FOS,CXCL2,HRAS,HSPA1A,HSPA5,HSP90AA1,ICAM1,IKBKB,IL1B,IL6,CXCL8,JAK2,JUN,MMP1,MMP3,MMP9,NFE2L2,NFKBIA,NOS3,PIK3CA,PPARG,MAPK1,MAPK3,MAPK8,RELA,CCL2,SELE,SELP,SRC,STAT3,TNF,TP53,VCAM1,CYCS,CCND1,CDK4,CDKN1A,CTNNB1,EP300,HIF1A,MYC,PTGS2,RAF1,RB1,STAT1,VEGFA,CCNA2,TGFB1,CDKN2A,EGFR,ITGB3,MDM2,EDN1,CXCL10,IRF1,MMP14,IFNG,IL4,EGF,PPARA,IL2,IL10,PTPN11,XIAP,ITGB1,NOS2,ACE,SERPINE1,IL1A,PARP1,CTSB,SPP1,APP,SOD1,CAV1 |
| hsa04933 | AGE-RAGE signaling pathway in diabetic complications | 31 | -51.31 | AKT1,CCND1,CASP3,CDK4,COL1A1,MAPK14,EDN1,HRAS,ICAM1,IL1A,IL1B,IL6,CXCL8,JAK2,JUN,MMP2,NOS3,SERPINE1,PIK3CA,MAPK1,MAPK3,MAPK8,RELA,CCL2,SELE,STAT1,STAT3,TGFB1,TNF,VCAM1,VEGFA,CAV1,CHUK,CTNNB1,FOS,HMOX1,HSP90AA1,IFNG,IKBKB,ITGB3,KDR,MMP9,NFE2L2,SRC,TP53 |
| hsa05205 | Proteoglycans in cancer | 36 | -49.86 | AKT1,CCND1,CASP3,CAV1,CDKN1A,COL1A1,MAPK14,CTNNB1,EGFR,ERBB2,ESR1,FGFR1,HIF1A,HRAS,IGF1R,IGF2,ITGB1,ITGB3,KDR,MDM2,MET,MMP2,MMP9,MYC,PIK3CA,PLAU,MAPK1,MAPK3,PTPN11,RAF1,SRC,STAT3,TGFB1,TNF,TP53,VEGFA,AR,CASP9,CHUK,EGF,EP300,HSP90AA1,IKBKB,MMP3,NFKBIA,PTEN,RB1,RELA,CDK4,CDKN2A,CXCL8,MMP1,BCL2L1,MAPK8,STAT1,XIAP,CCNA2,FOS,ICAM1,IL2,IL6,JUN,MMP7,TERT,ESR2,CASP8,IRF1,PTGS2,SPP1,KIT,PGR,IL10,SLC2A4,IGFBP3,IL1A,SERPINE1,HMOX1,NFE2L2,CYCS,DNMT1,ABCB1 |
| hsa04151 | PI3K-Akt signaling pathway | 38 | -44.18 | AKT1,CCND1,BCL2L1,CASP9,CDK4,CDKN1A,CHUK,COL1A1,EGF,EGFR,ERBB2,FGFR1,HRAS,HSP90AA1,IGF1R,IGF2,IKBKB,IL2,IL4,IL6,ITGB1,ITGB3,JAK2,KDR,KIT,MDM2,MET,MYC,NOS3,PIK3CA,MAPK1,MAPK3,PTEN,RAF1,RELA,SPP1,TP53,VEGFA,CASP3,MAPK14,FOS,HSPA1A,HSPB1,IL1A,IL1B,JUN,MAPK8,TGFB1,TNF,PTPN11 |
| hsa05235 | PD-L1 expression and PD-1 checkpoint pathway in cancer | 22 | -33.72 | AKT1,CHUK,MAPK14,EGF,EGFR,FOS,HIF1A,HRAS,IFNG,IKBKB,JAK2,JUN,NFKBIA,PIK3CA,MAPK1,MAPK3,PTEN,PTPN11,RAF1,RELA,STAT1,STAT3,COL1A1,EDN1,MMP1,MMP2,MMP9,NOS2,NOS3,MAPK8,SRC,TGFB1,VEGFA,XIAP,AR,CCND1,CYP3A4,ESR1,ESR2,HSP90AA1,MYC,PGR,PPARA,RB1,CAV1,CTNNB1,ERBB2,IGF1R,ITGB1,ITGB3,KDR,MET,SPP1,CD40LG,CDK4,IL2,IL4,IL10,TNF,HMOX1,NFE2L2,SOD1,BCL2L1,IL6,IRF1,CCNA2,KIT,MPO,HSPA1A,FGFR1,TP53,CASP9,HSPB1,PTGS2,CDKN1A,EP300,CXCL2,CXCL8,CXCL10,CCL2,IGFBP3,MMP14,CTSB,CASP1,SELP,CASP3,ICAM1,SERPINE1,RUNX2,SLC2A4,GJA1,APP |
| hsa05222 | Small cell lung cancer | 21 | -31.36 | AKT1,XIAP,CCND1,BCL2L1,CASP3,CASP9,CDK4,CDKN1A,CHUK,IKBKB,ITGB1,MYC,NFKBIA,NOS2,PIK3CA,PTEN,PTGS2,RB1,RELA,TP53,CYCS,CASP8,CDKN2A,ERBB2,MDM2,MAPK1,MAPK3,IGFBP3,SERPINE1,MAPK8,CAV1,CD40LG,ICAM1,CASP1,MAPK14,HSPA5,SOD1,TNF,NFE2L2,EP300,PPARG |
| hsa04066 | HIF-1 signaling pathway | 21 | -29.66 | AKT1,CDKN1A,EDN1,EGF,EGFR,EP300,ERBB2,HIF1A,HMOX1,IFNG,IGF1R,IL6,NOS2,NOS3,SERPINE1,PIK3CA,MAPK1,MAPK3,RELA,STAT3,VEGFA,CTNNB1,FGFR1,MET,SRC,KDR |
| hsa04630 | JAK-STAT signaling pathway | 20 | -23.91 | AKT1,CCND1,BCL2L1,CDKN1A,EGF,EGFR,EP300,HRAS,IFNG,IL2,IL4,IL6,IL10,JAK2,MYC,PIK3CA,PTPN11,RAF1,STAT1,STAT3 |
| hsa05144 | Malaria | 14 | -22.36 | CD40LG,ICAM1,IFNG,IL1B,IL6,CXCL8,IL10,MET,CCL2,SELE,SELP,TGFB1,TNF,VCAM1,FOS,CXCL2,IL1A,JUN,MMP1,MMP3,VEGFA,IL2,IL4,RELA,STAT1,STAT3,CXCL10,ITGB3,KIT |
| hsa04064 | NF-kappa B signaling pathway | 16 | -20.94 | PARP1,XIAP,BCL2L1,CD40LG,CHUK,CXCL2,ICAM1,IKBKB,IL1B,CXCL8,NFKBIA,PLAU,PTGS2,RELA,TNF,VCAM1,AKT1,CCND1,CASP3,CASP8,MAPK14,CTNNB1,IL6,PPARA,MAPK8,EGFR,JUN,MET,PTPN11,SRC,CASP1,CASP9,HSPA1A,CYCS,COL1A1,HSPB1,IFNG,IL10,NOS2,PIK3CA,TGFB1,CXCL10 |
| hsa05202 | Transcriptional misregulation in cancer | 18 | -19.48 | BCL2L1,RUNX2,CCNA2,CDKN1A,IGF1R,IGFBP3,IL6,CXCL8,MDM2,MET,MMP3,MMP9,MPO,MYC,PLAU,PPARG,RELA,TP53 |
| hsa04931 | Insulin resistance | 14 | -17.26 | AKT1,IKBKB,IL6,NFKBIA,NOS3,PIK3CA,PPARA,MAPK8,PTEN,PTPN11,RELA,SLC2A4,STAT3,TNF,CHUK,JAK2,PARP1,COL1A1,MAPK14,ACE,MMP2,MMP9,TGFB1 |
| hsa05216 | Thyroid cancer | 9 | -13.89 | CCND1,CDKN1A,CTNNB1,HRAS,MYC,PPARG,MAPK1,MAPK3,TP53,CDK4,CDKN2A,EGFR,RB1 |
| hsa04217 | Necroptosis | 13 | -13.4 | PARP1,XIAP,CASP1,CASP8,HSP90AA1,IFNG,IL1A,IL1B,JAK2,MAPK8,STAT1,STAT3,TNF |
| hsa04137 | Mitophagy - animal | 8 | -9.5 | BCL2L1,HIF1A,HRAS,JUN,MAPK8,RELA,SRC,TP53,CCND1,CDK4,ERBB2,ITGB1 |
| hsa04670 | Leukocyte transendothelial migration | 9 | -9.28 | MAPK14,CTNNB1,ICAM1,ITGB1,MMP2,MMP9,PIK3CA,PTPN11,VCAM1,CAV1,MET,SRC |
| hsa04211 | Longevity regulating pathway | 7 | -7.31 | AKT1,HRAS,IGF1R,PIK3CA,PPARG,RELA,TP53,HSPA1A,SOD1,CCND1,CCNA2,SLC2A4,PTGS2,MAPK8,CYCS |
| hsa05410 | Hypertrophic cardiomyopathy | 7 | -7.28 | ACE,EDN1,IL6,ITGB1,ITGB3,TGFB1,TNF,CTNNB1,GJA1,COL1A1,SPP1 |
| hsa04350 | TGF-beta signaling pathway | 7 | -7.12 | EP300,IFNG,MYC,MAPK1,MAPK3,TGFB1,TNF |
