# Supplementary material for: Exploring the efficacy and molecular mechanism of Danhong injection comprehensively in the treatment of idiopathic pulmonary fibrosis by combining meta-analysis, network pharmacology, and molecular docking methods
Source: Medicine (Baltimore). 2024 May 10;103(19):e38133. doi: 10.1097/MD.0000000000038133 (PMC11081554; doi:10.1097/MD.0000000000038133)

Study or Subgroup	Experimental Mean	Experimental SD	Experimental Total	Control Mean	Control SD	Control Total	Weight	Mean Difference IV, Random, 95% CI
Lin 2016	92.45	31.13	35	139.78	32.74	35	24.4%	-47.33 [-62.30, -32.36]
Wang 2020	98.13	17.38	60	110.52	22.19	60	27.4%	-12.39 [-19.52, -5.26]
Wu 2018	95.25	30.63	30	107.36	35.25	30	23.6%	-12.11 [-28.82, 4.60]
Zhao 2016	91.53	32.46	40	136.25	33.47	40	24.6%	-44.72 [-59.17, -30.27]
**Total (95% CI)**			**165**			**165**	**100.0%**	**-28.81 [-48.52, -9.11]**

Heterogeneity: $Tau^2 = 355.89$; $Chi^2 = 28.82$, $df = 3$ ($P < 0.00001$); $I^2 = 90\%$
Test for overall effect: $Z = 2.87$ ($P = 0.004$)

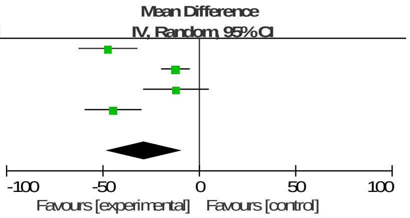

Supplement: Supplementary file 10 [file medi-103-e38133-s011.doc]
