# Supplementary material for: Exploring the efficacy and molecular mechanism of Danhong injection comprehensively in the treatment of idiopathic pulmonary fibrosis by combining meta-analysis, network pharmacology, and molecular docking methods
Source: Medicine (Baltimore). 2024 May 10;103(19):e38133. doi: 10.1097/MD.0000000000038133 (PMC11081554; doi:10.1097/MD.0000000000038133)

Study or Subgroup	Experimental Mean	Experimental SD	Experimental Total	Control Mean	Control SD	Control Total	Weight	Mean Difference IV, Random, 95% CI
Lin 2016	112.06	33.92	35	156.48	31.64	35	23.5%	-44.42 [-59.79, -29.05]
Wang 2020	103.68	24.17	60	124.65	28.93	60	29.3%	-20.97 [-30.51, -11.43]
Wu 2018	110.42	31.31	30	156.24	32.14	30	22.9%	-45.82 [-61.88, -29.76]
Zhao 2016	110.42	32.76	40	149.26	33.48	40	24.4%	-38.84 [-53.36, -24.32]
**Total (95% CI)**			**165**			**165**	**100.0%**	**-36.52 [-49.71, -23.32]**

Heterogeneity: Tau² = 131.21; Chi² = 11.30, df = 3 (P = 0.01); I² = 73%
Test for overall effect: Z = 5.42 (P < 0.00001)

Mean Difference
IV, Random, 95% CI

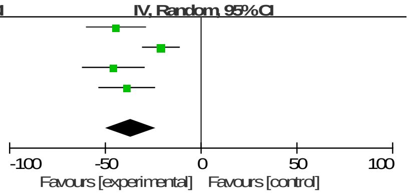

Supplement: Supplementary file 11 [file medi-103-e38133-s012.doc]
