# Supplementary material for: Exploring the efficacy and molecular mechanism of Danhong injection comprehensively in the treatment of idiopathic pulmonary fibrosis by combining meta-analysis, network pharmacology, and molecular docking methods
Source: Medicine (Baltimore). 2024 May 10;103(19):e38133. doi: 10.1097/MD.0000000000038133 (PMC11081554; doi:10.1097/MD.0000000000038133)

	Experimental			Control				Mean Difference
**Study or Subgroup**	**Mean**	**SD**	**Total**	**Mean**	**SD**	**Total**	**Weight**	**IV, Fixed, 95% CI**
Lin 2016	87.42	19.24	35	109.81	24.13	35	16.8%	-22.39 [-32.61, -12.17]
Wang 2020	84.3	17.09	60	96.18	15.81	60	50.5%	-11.88 [-17.77, -5.99]
Wu 2018	87.62	18.42	30	100.56	20.95	30	17.6%	-12.94 [-22.92, -2.96]
Zhao 2016	85.38	22.46	40	108.57	26.35	40	15.2%	-23.19 [-33.92, -12.46]
**Total (95% CI)**			**165**			**165**	**100.0%**	**-15.55 [-19.73, -11.36]**

Heterogeneity: $Chi^2 = 5.42$, df = 3 (P = 0.14); $I^2 = 45\%$
Test for overall effect: Z = 7.28 (P < 0.00001)

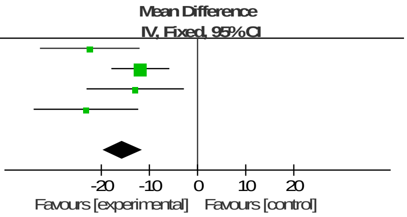

Supplement: Supplementary file 12 [file medi-103-e38133-s013.doc]
