# Supplementary material for: Exploring the efficacy and molecular mechanism of Danhong injection comprehensively in the treatment of idiopathic pulmonary fibrosis by combining meta-analysis, network pharmacology, and molecular docking methods
Source: Medicine (Baltimore). 2024 May 10;103(19):e38133. doi: 10.1097/MD.0000000000038133 (PMC11081554; doi:10.1097/MD.0000000000038133)

	Experimental			Control				Mean Difference	Mean Difference
Study or Subgroup	Mean	SD	Total	Mean	SD	Total	Weight	IV, Fixed, 95% CI	IV, Fixed, 95% CI
Lin 2016	91.78	19.27	35	129.34	17.24	35	52.1%	-37.56 [-46.13, -28.99]	
Zhao 2016	91.86	16.73	40	124.46	23.47	40	47.9%	-32.60 [-41.53, -23.67]	
**Total (95% CI)**			**75**			**75**	**100.0%**	**-35.18 [-41.37, -29.00]**	

Heterogeneity: Chi² = 0.62, df = 1 (P = 0.43); I² = 0%

Test for overall effect: Z = 11.15 (P < 0.00001)

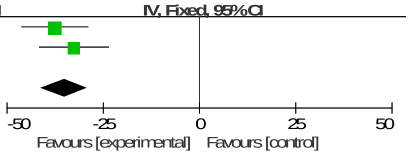

Supplement: Supplementary file 13 [file medi-103-e38133-s014.doc]
