# Supplementary material for: Exploring the efficacy and molecular mechanism of Danhong injection comprehensively in the treatment of idiopathic pulmonary fibrosis by combining meta-analysis, network pharmacology, and molecular docking methods
Source: Medicine (Baltimore). 2024 May 10;103(19):e38133. doi: 10.1097/MD.0000000000038133 (PMC11081554; doi:10.1097/MD.0000000000038133)

Study or Subgroup	Experimental Mean	SD	Total	Control Mean	SD	Total	Weight	Mean Difference IV, Fixed, 95% CI
Lin 2016	11.79	3.75	35	16.12	4.16	35	46.4%	-4.33 [-6.19, -2.47]
Zhao 2016	10.98	4.54	40	16.37	3.22	40	53.6%	-5.39 [-7.11, -3.67]
**Total (95% CI)**			**75**			**75**	**100.0%**	**-4.90 [-6.16, -3.64]**

Heterogeneity: $Chi^2 = 0.67$, $df = 1$ ($P = 0.41$); $I^2 = 0\%$
Test for overall effect: $Z = 7.60$ ($P < 0.00001$)

Mean Difference
IV, Fixed, 95% CI

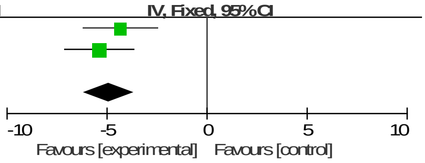

Supplement: Supplementary file 14 [file medi-103-e38133-s015.doc]
